# Supplementary material for: Thioester synthesis through geoelectrochemical CO2 fixation on Ni sulfides
Source: Commun Chem. 2021 Mar 17;4:37. doi: 10.1038/s42004-021-00475-5 (PMC9814748; doi:10.1038/s42004-021-00475-5)
Supplement: Supplementary file 1 — Supplementary Information [file 42004_2021_475_MOESM1_ESM.pdf]

Supplementary Information for

Thioester synthesis through geoelectrochemical CO_2 fixation on Ni sulfides

Norio Kitadai*, Ryuhei Nakamura, Masahiro Yamamoto, Satoshi Okada, Wataru Takahagi, Yuko Nakano, Yoshio Takahashi, Ken Takai, and Yoshi Oono

Table of contents

Supplementary Figs. 1–28
Supplementary Tables 1–5
References (1–8)

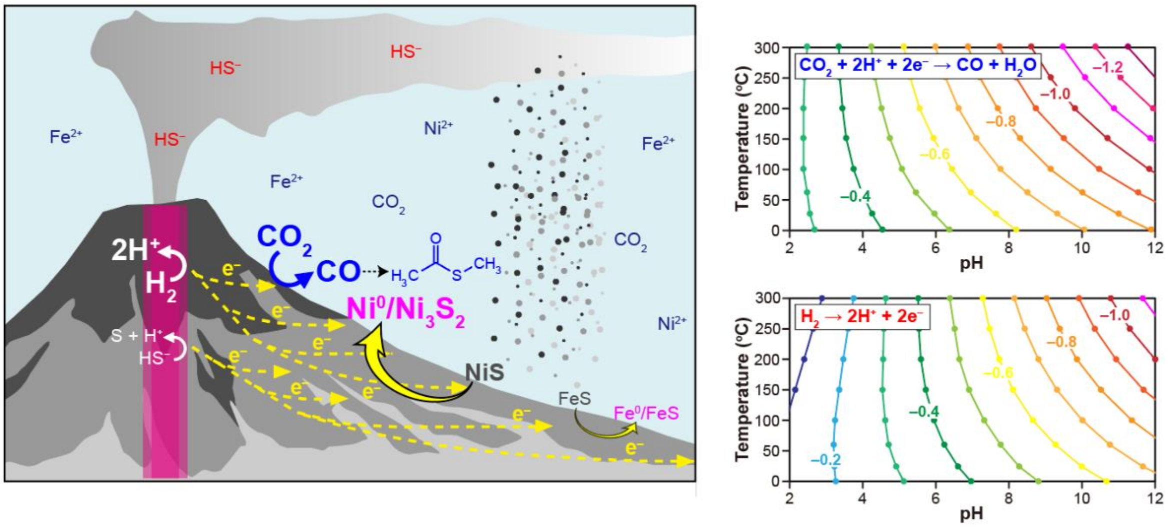

Supplementary Fig. 1 Geoelectricity-driven abiotic CO_2 fixation in an early ocean hydrothermal system. (left) oxidation of reductive hydrothermal fluid chemicals (for example, H_2 and H_2S) at the fluid-chimney interface provided sustained negative electric potentials at the ancient seawater-chimney interface, where metal sulfide precipitates were electroreduced to metastable sulfide/metal composites, thereby drastically enhancing their capabilities of driving nonenzymatic CO_2 fixation and the subsequent protometabolic reactions as demonstrated in the present study and by Kitadai et al.[1]. (right) Thermodynamic calculation for the H^+/H_2 and the CO_2/CO redox potentials (V_{SHE}) as a function of temperature and pH at 500 bar indicates that H_2 oxidation in hot and alkaline pH conditions readily generate negative electric potentials favorable for the CO_2-to-CO conversion in cool (0–50 °C) and slightly acidic (pH 6–7) ancient seawater[2]. In this calculation, 1 mmol kg^{-1} H_2 is considered because it is a typical H_2 concentration in fluids from the present-day serpentine-hosted hydrothermal systems[3,4]. The CO_2/CO activity ratio is set to one. Equilibrium calculation with this ratio gives the potential conditions where CO_2 and CO are equally stable. A ten-fold change in the molecular species concentration changes the redox potential by ± ~30 mV at 25°C and by ± ~40 mV at 150°C.

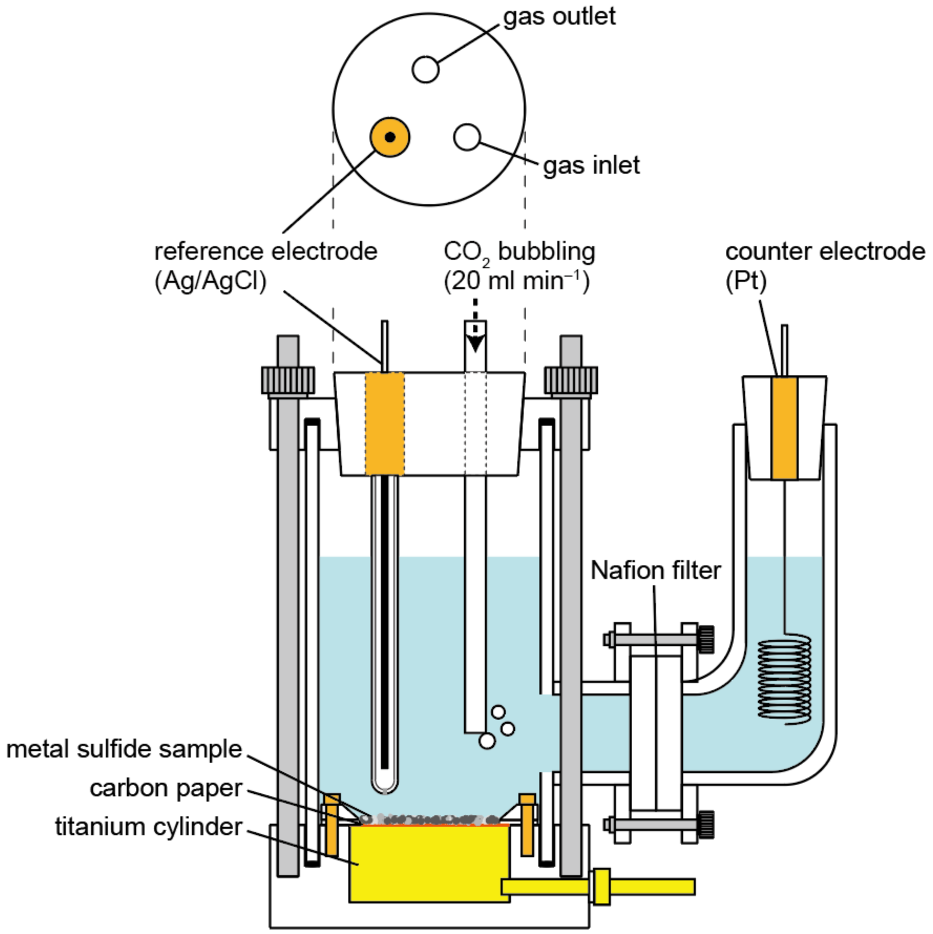

Supplementary Fig. 2 A schematic of the electrochemical cell. The cell is made of a Pyrex glass tube sandwiched between a polyoxymethylene (POM) cap and basement that were tightened together with stainless screws and knurled nuts. The cell has two compartments: a large working electrode side (~100 ml) and a small counter electrode side (~15 ml) that are separated by a proton exchange membrane (Nafion 117; DuPont). On the working electrode side, a titanium cylinder (purity; 99.5%) is placed at the center of the POM basement, and is coated with carbon paper (5.7 cm^2) with a silicon and POM packings. An Ag/AgCl electrode (in saturated KCl) is used as the reference, and is fixed at a distance of less than 5 mm from the working electrode to reduce solution resistance. On the counter side, a platinum coil is inserted into the glass tube, and is used as the counter electrode.

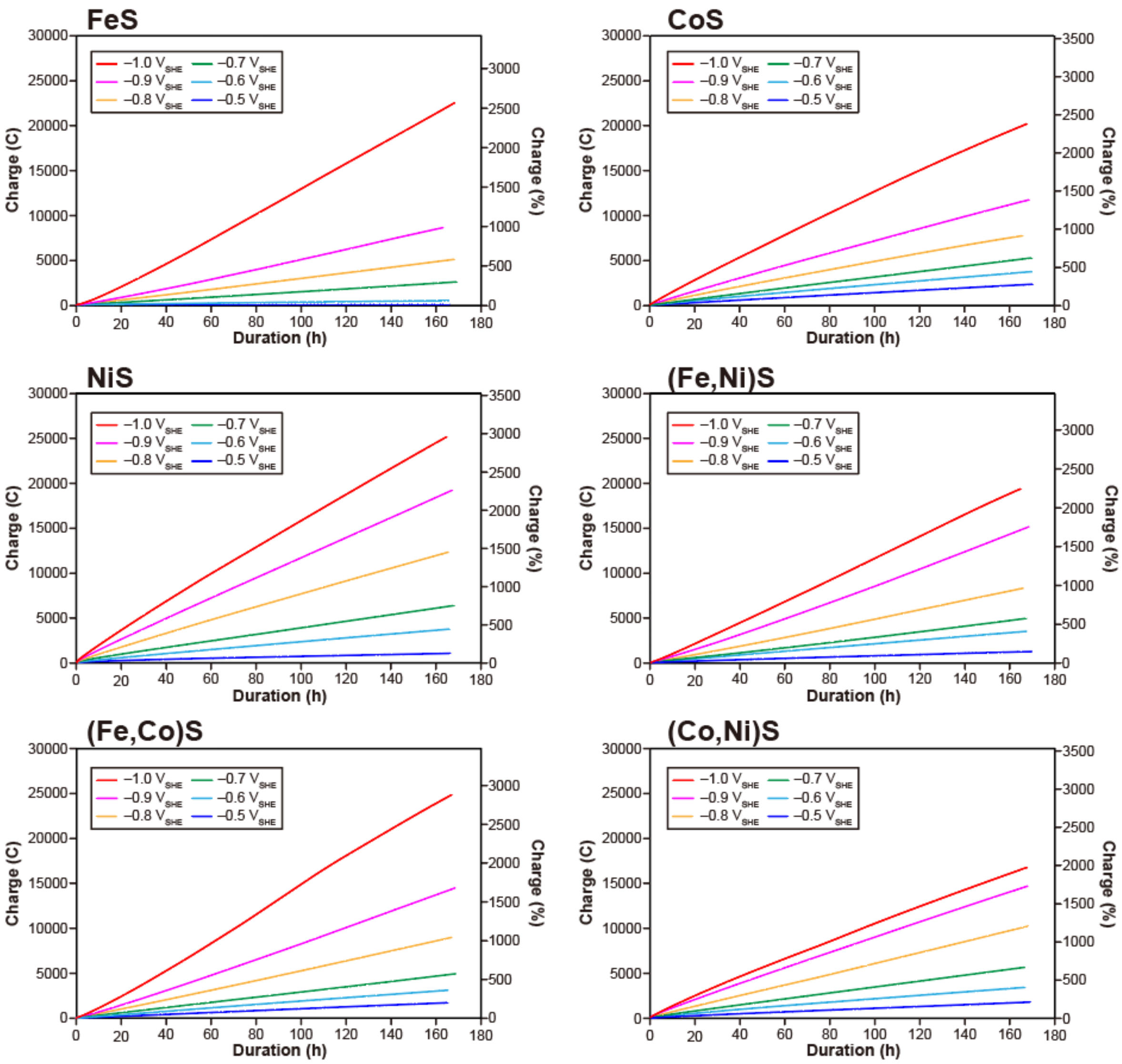

Supplementary Fig. 3 Total charges built up during the electrolysis. The percentages shown at the right axis were calculated relative to the charges required for the complete electroreduction of respective sulfide samples (400 mg) to the corresponding zerovalent metals. In practice, a fraction of metal sulfide floated with electrochemically generated gas bubbles and deposited onto a cell compartment out of the electrode. The percentage remaining on the electrode after the 7-day electrolysis was around 30% at –1.0 V_{SHE} for the case of NiS.

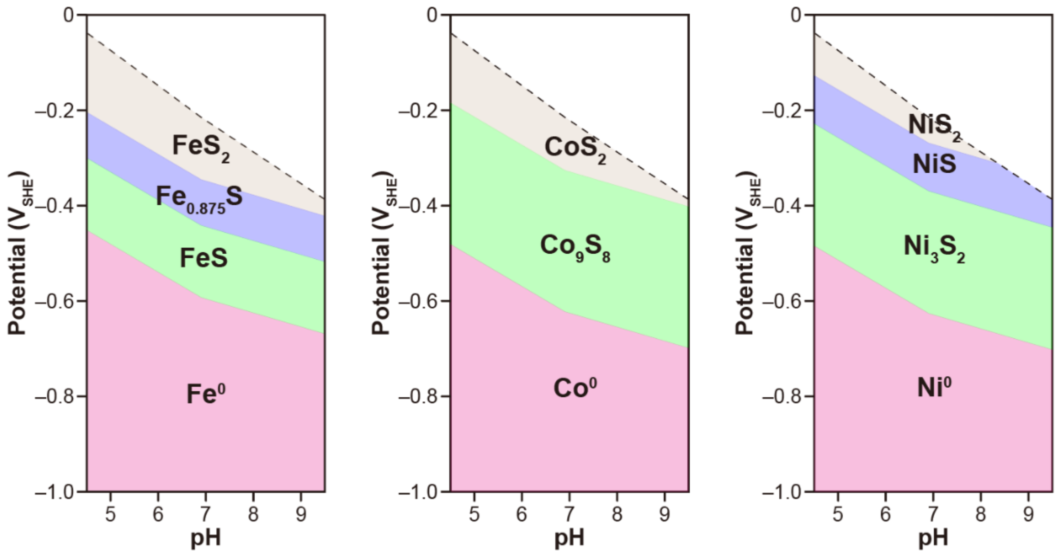

Supplementary Fig. 4 E_H-pH diagrams for the sulfide/metal system of Fe, Co, and Ni.

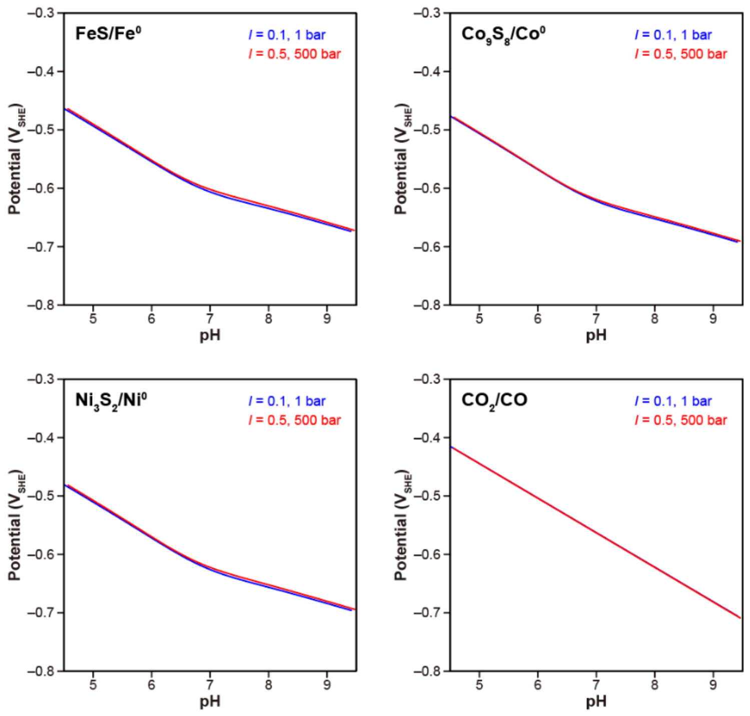

Supplementary Fig. 5 Comparisons of the sulfide/metal and CO_2/CO redox potentials at the ionic strength (I) of 0.1 and 1 bar (blue) with those at I = 0.5 and 500 bar (red). In both cases, temperature was set to 25°C.

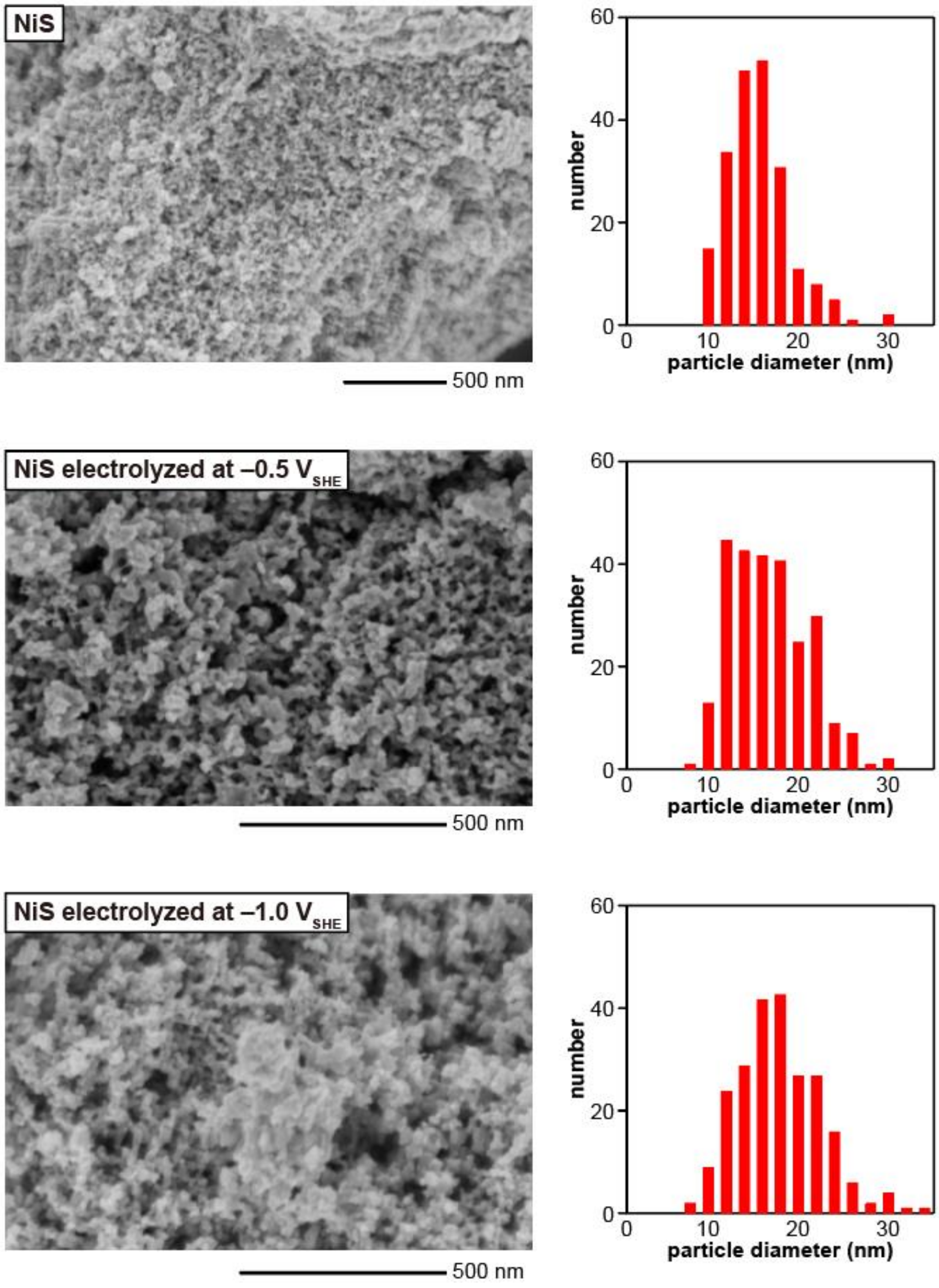

Supplementary Fig. 6 SEM images and particle size distributions of NiS before and after the electrolysis. The average particle diameters of NiS, NiS electrolyzed at –0.5 V_{SHE}, and that at –1.0 V_{SHE} were estimated with standard deviations to be 15 ± 4 nm (N = 209), 16 ± 4 nm (N = 259), 17 ± 5 nm (N = 233), respectively. Scale bars represent 500 nm.

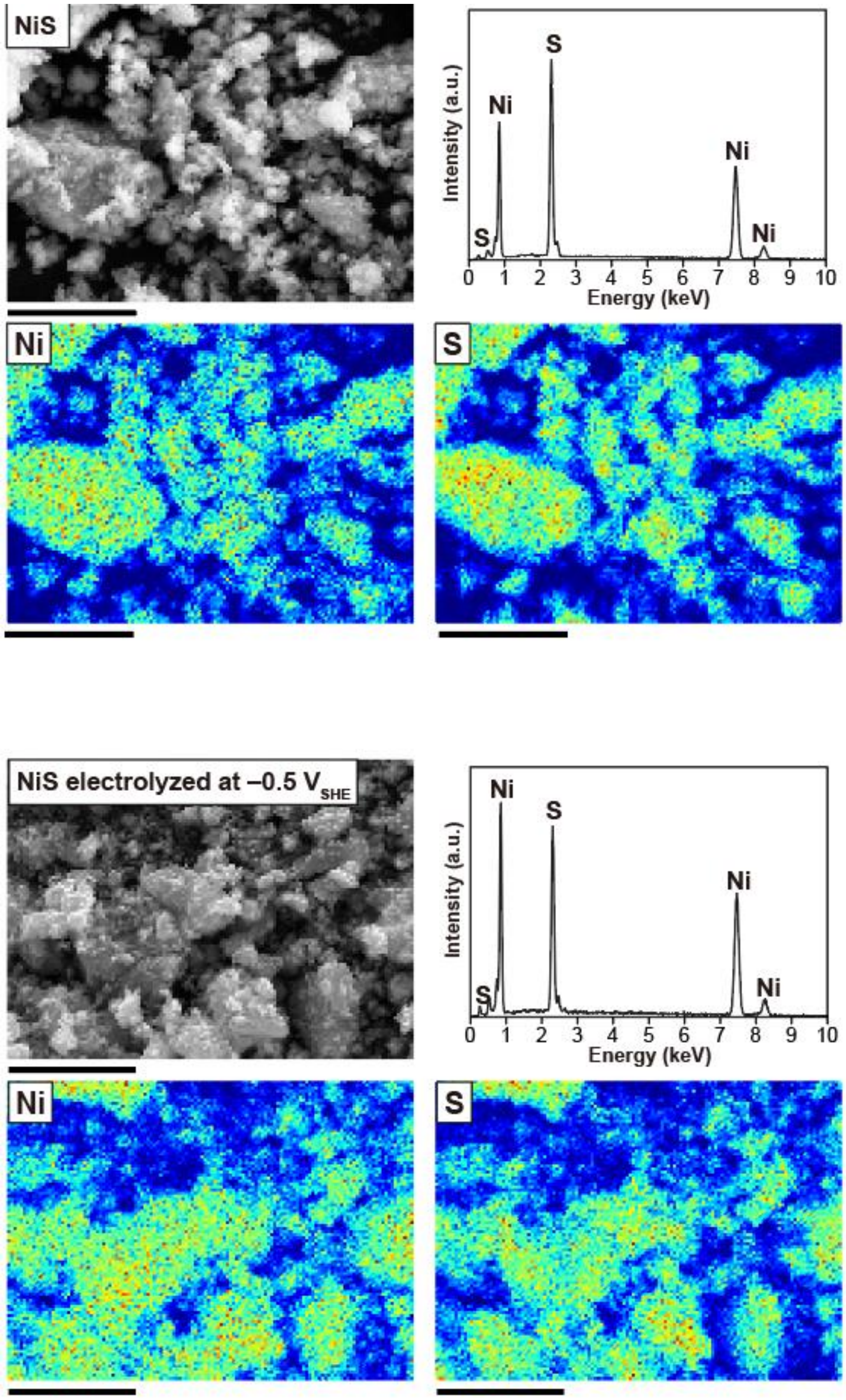

Supplementary Fig. 7 EDX mapping data of sulfide samples. Scale bars represent 10 μm.

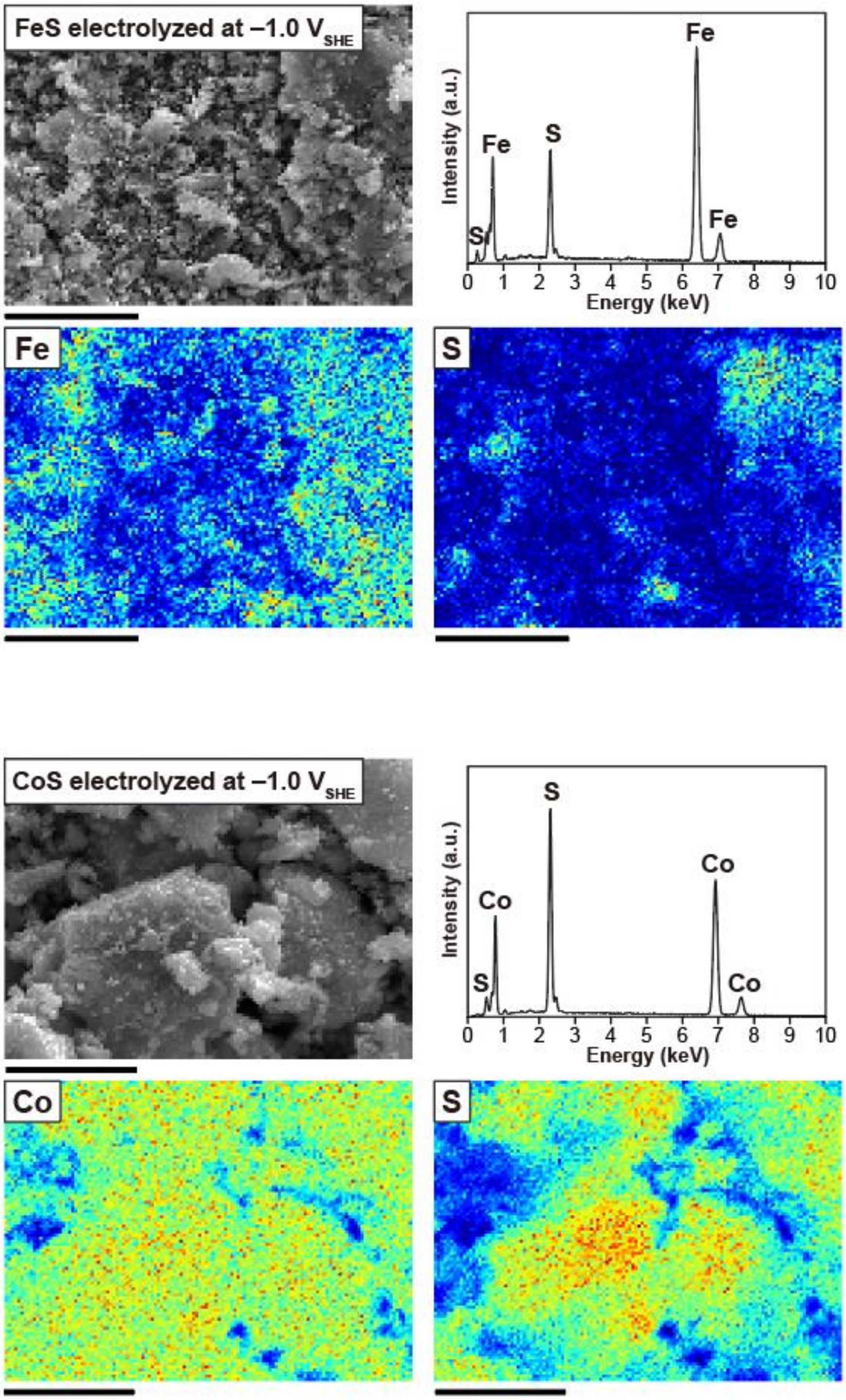

Supplementary Fig. 7 continued.

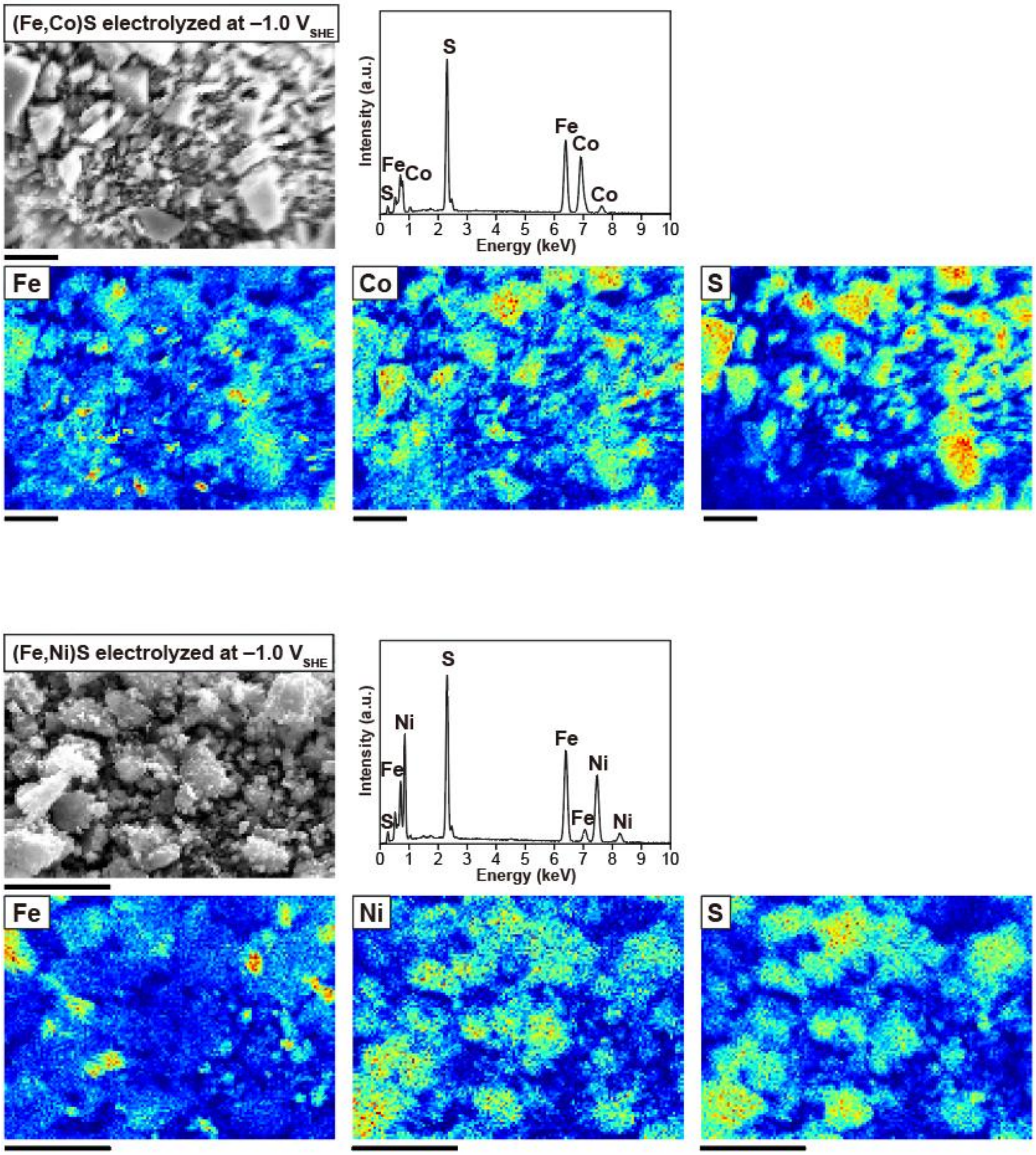

Supplementary Fig. 7 continued.

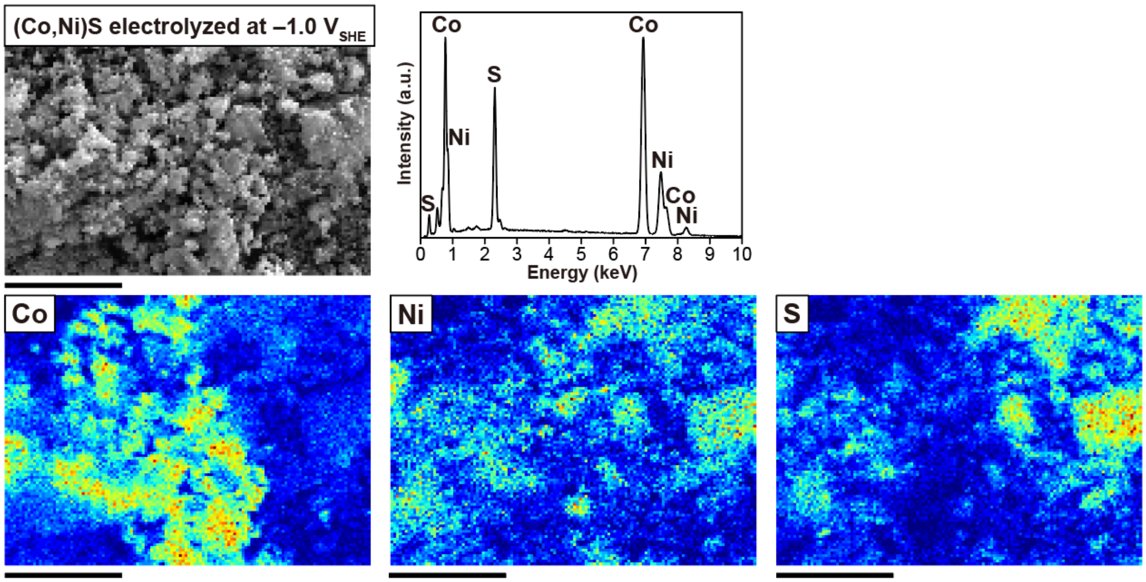

Supplementary Fig. 7 continued.

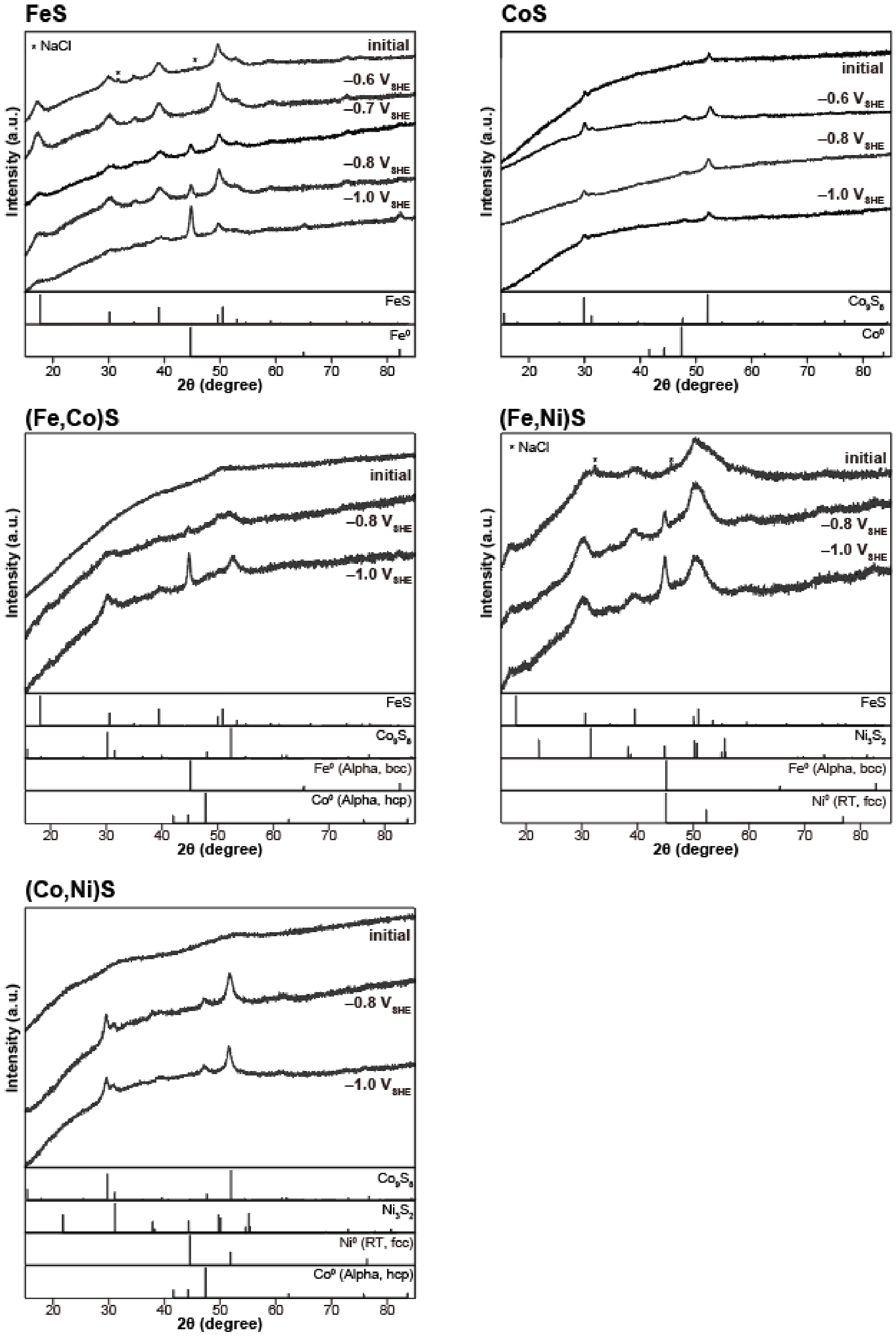

Supplementary Fig. 8 XRD patterns of metal sulfides before and after the electrolysis.

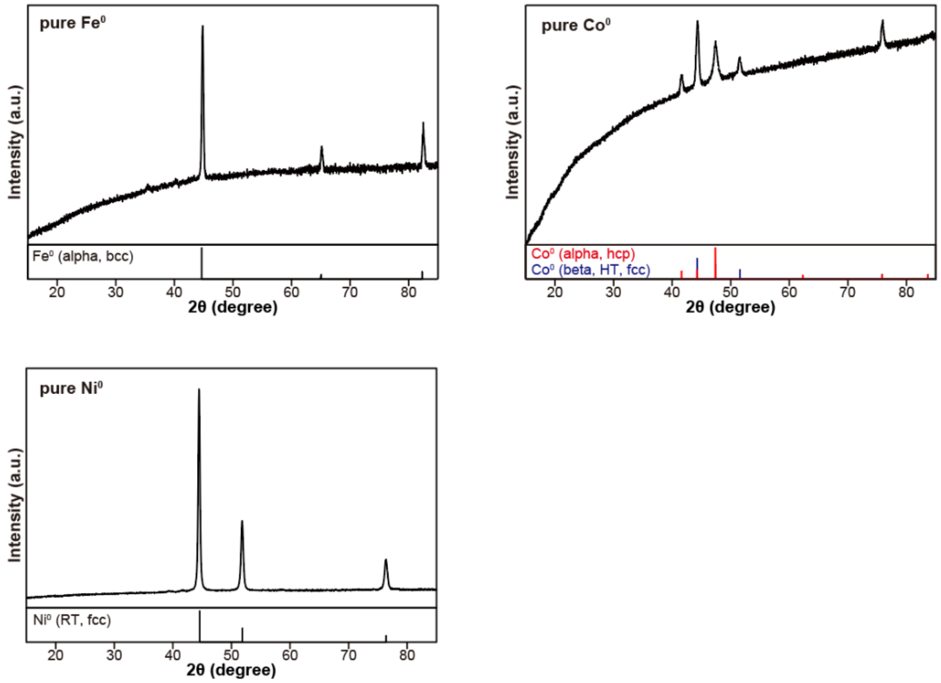

Supplementary Fig. 9 XRD patterns of pure metals used in this study.

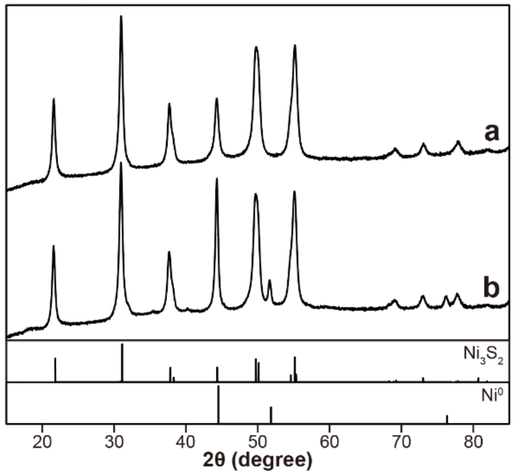

Supplementary Fig. 10 Comparison of the XRD pattern between (**a**) the NiS_PERM prepared at –1.0 V_{SHE} and (**b**) a mixture of pure Ni^0 and Ni_3S_2 (Ni in Ni^0 : Ni in Ni_3S_2 = 12.1 : 87.9). Pure Ni^0 was obtained commercially (EM Japan), whereas Ni_3S_2 was prepared by the NiS electroreduction at –0.5 V_{SHE} for 7 days (see Methods).

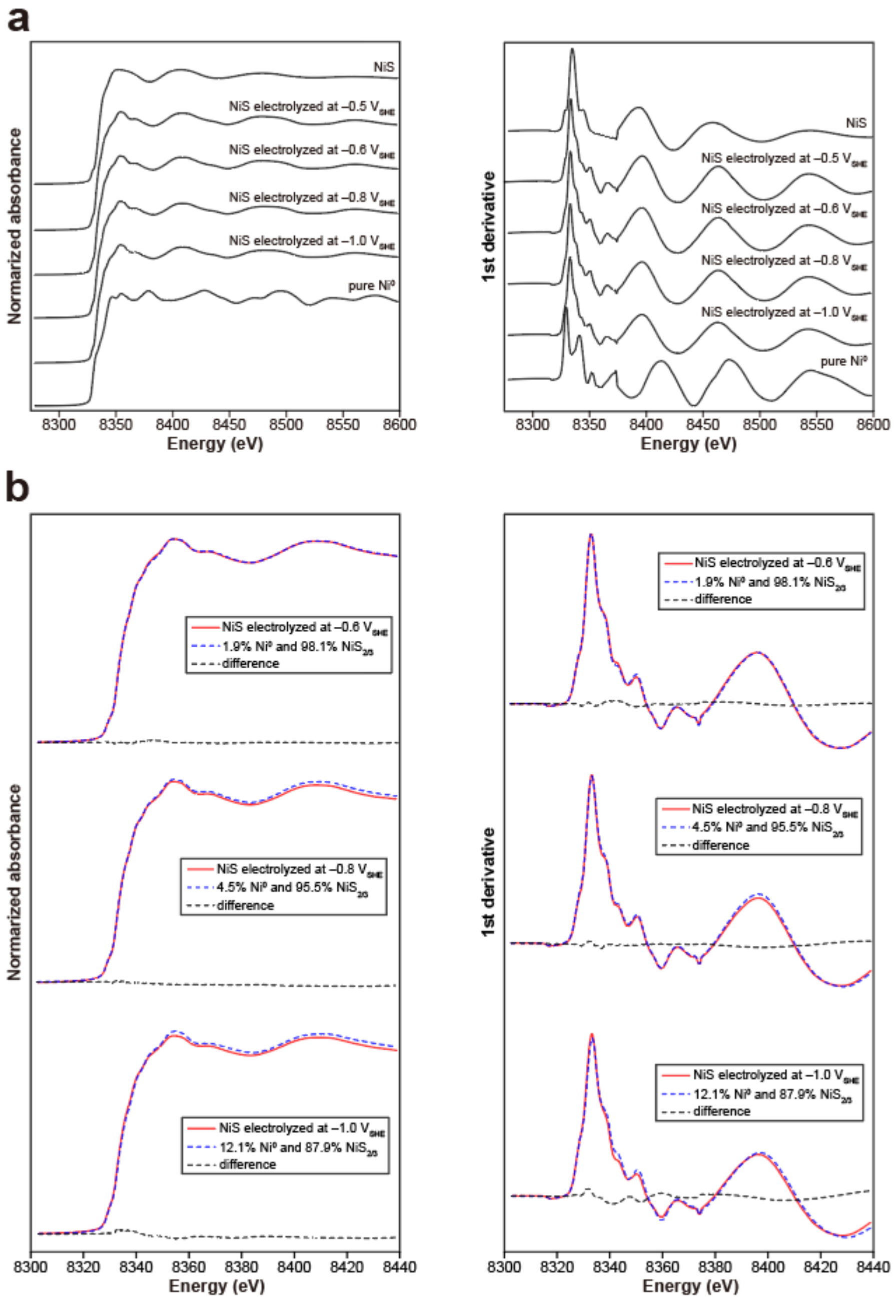

Supplementary Fig. 11 Normalized (left) and first derivative (right) nickel K-edge XANES spectra of NiS samples (**a**) and spectral fitting results (**b**). Fitting was conducted with the linear combination of pure Ni^0 and the NiS electrolyzed at –0.5 V_{SHE} corresponding to heazlewoodite (Ni_3S_2). See Materials and Methods for details of the fitting analysis.

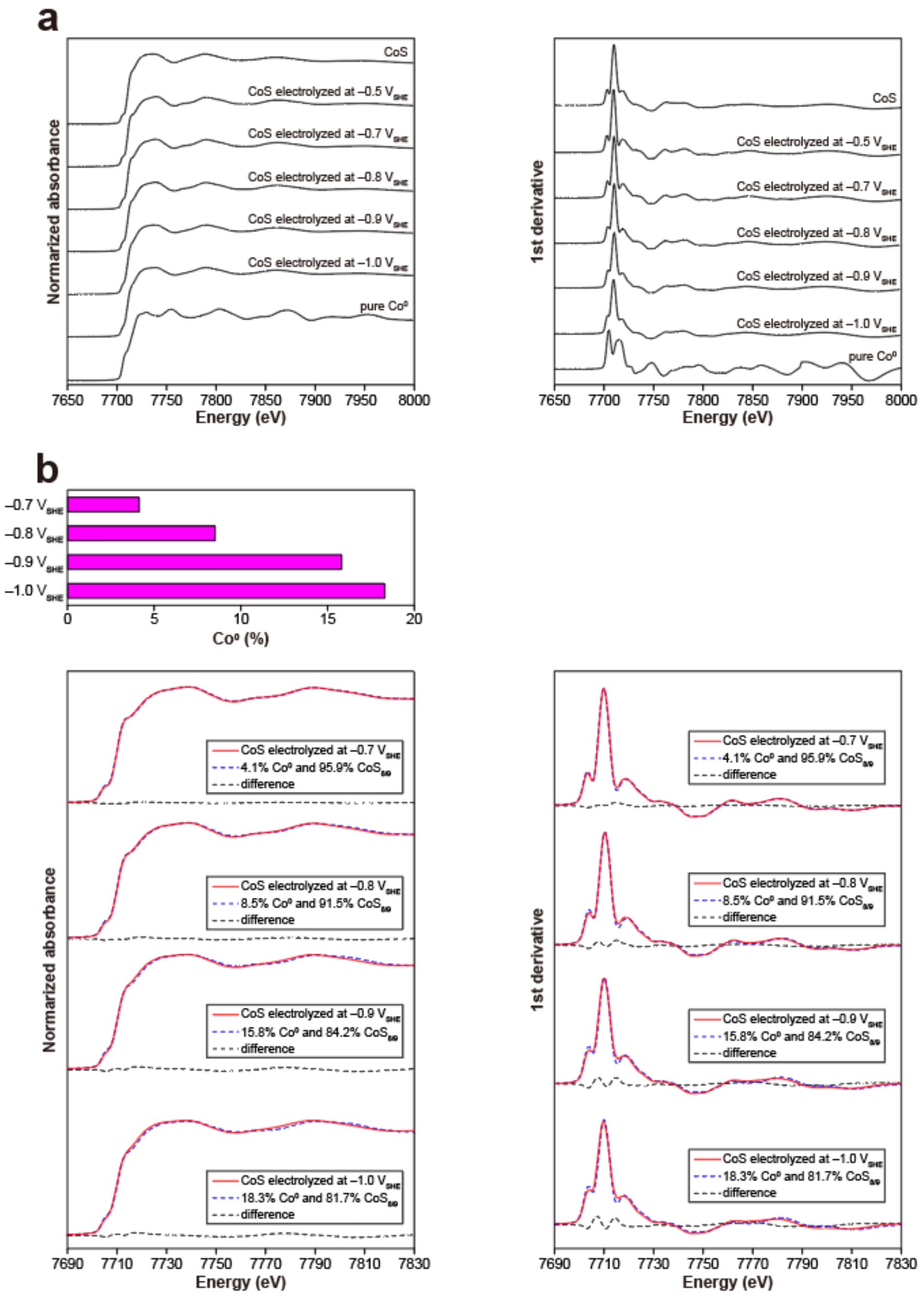

Supplementary Fig. 12 Normalized (left) and first derivative (right) cobalt K-edge XANES spectra of CoS samples (**a**) and spectral fitting results (**b**). Fitting was conducted with the linear combination of pure Co^0 and the CoS electrolyzed at –0.5 V_{SHE}.

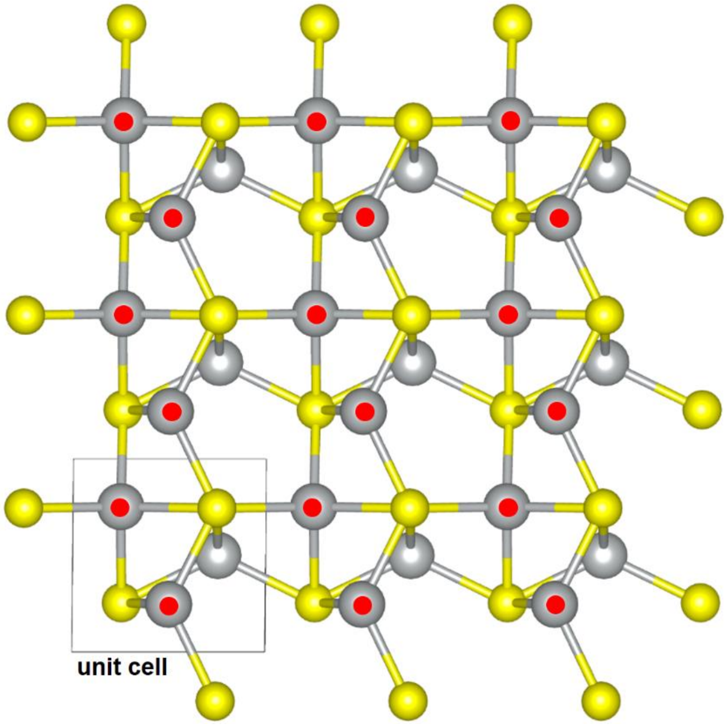

Supplementary Fig. 13 Surface of Ni_3S_2 depicted based on the reported crystal structure[5]. Ni and S atoms are shown with gray and yellow spheres, respectively, and the Ni atoms exposed to the surface are marked with red dots. Two Ni atoms are present on a unit cell surface area of 16.66 Å^2, which corresponds to 2.0×10^{-5} mole of surface Ni atoms per m^2. If we assume the electrolyzed NiS particle to be a sphere of diameter 17 nm on the average (Supplementary Fig. 6), 180 ± 40 μmol g^{-1} of the adsorbed CO (Fig. 2a) corresponds to one CO molecule per 8 surface Ni atoms.

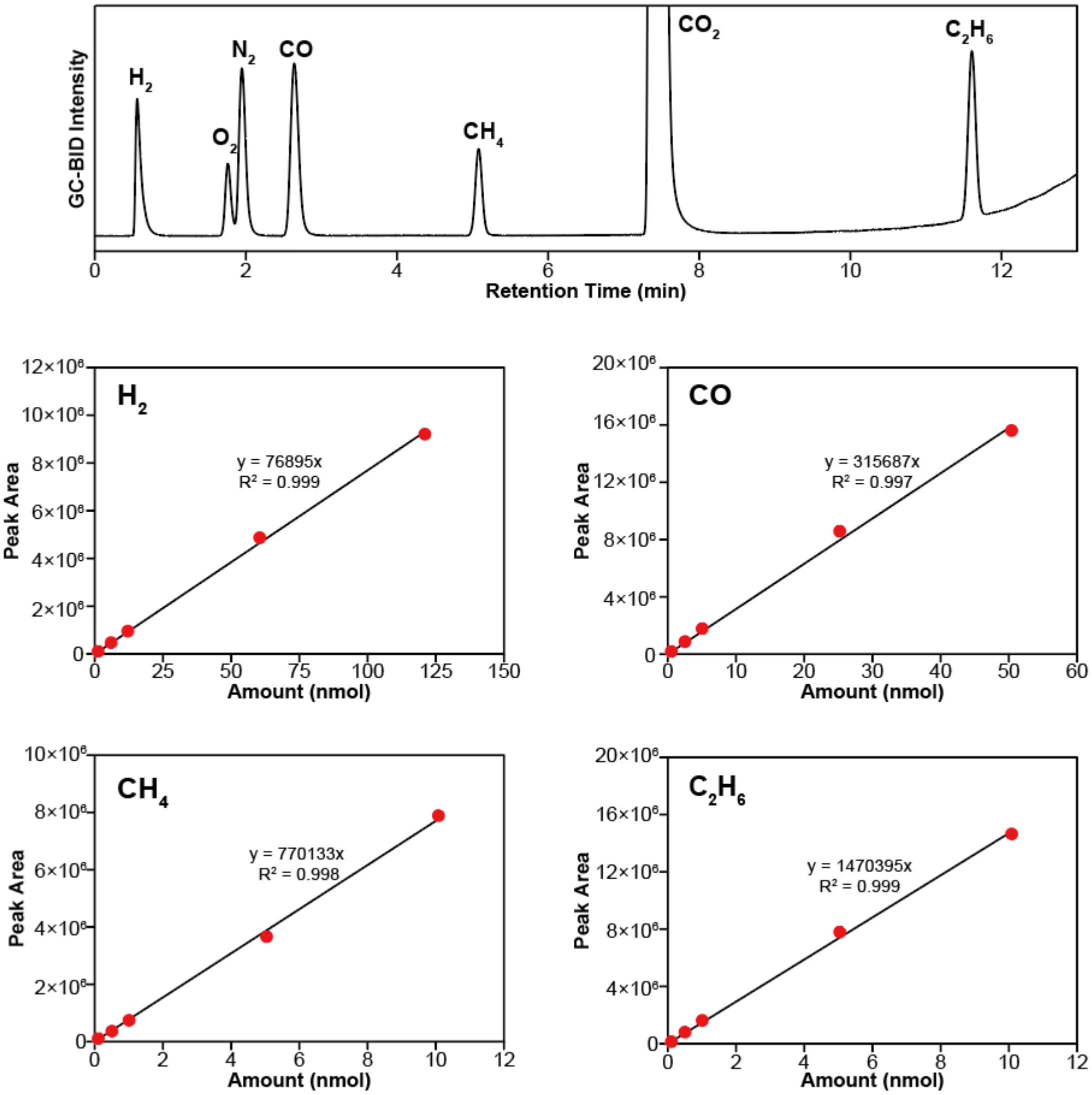

Supplementary Fig. 14 Calibration curves for H_2, CO, CH_4, and C_2H_6 by the GC-BID detector system.

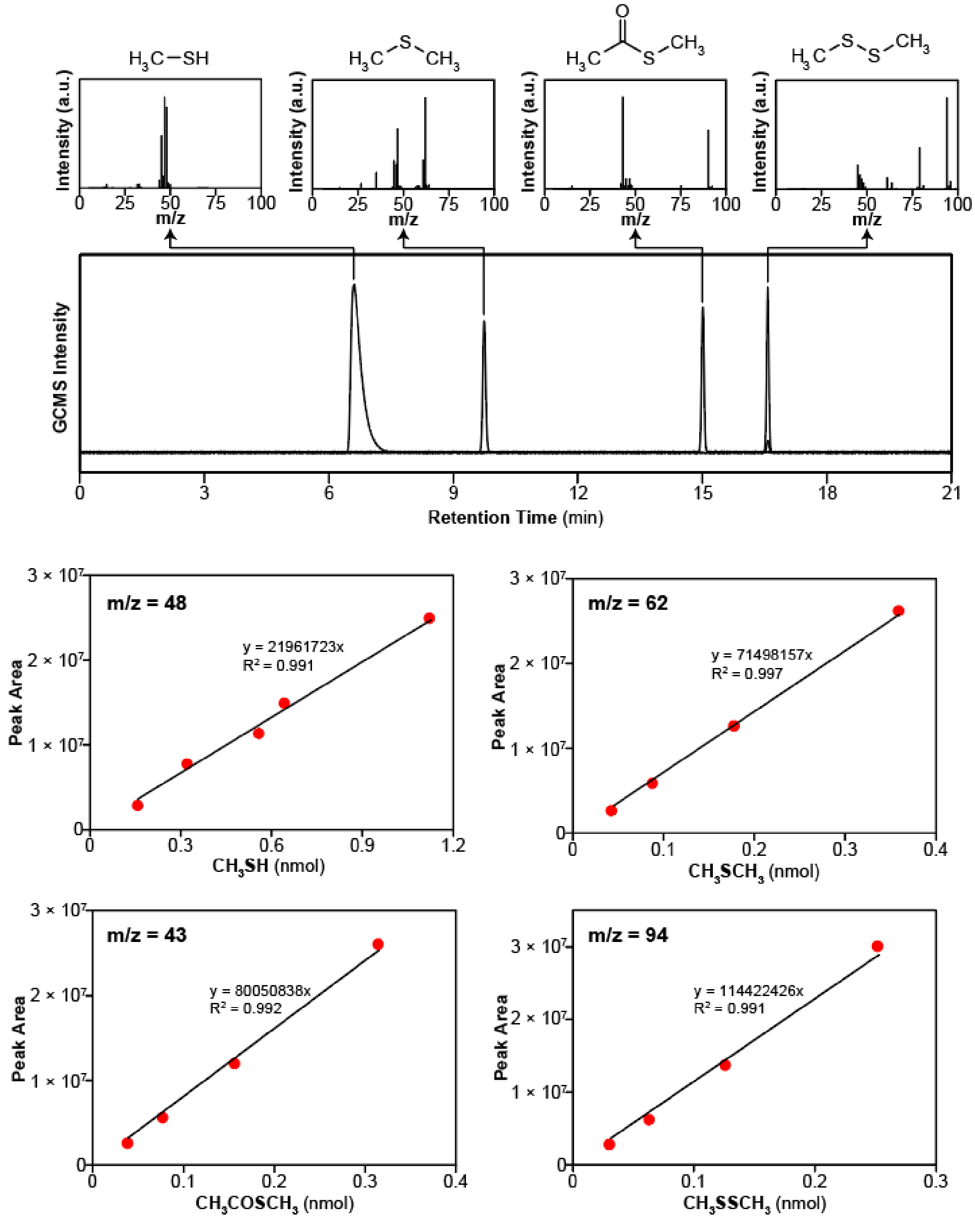

Supplementary Fig. 15 Calibration curves for organosulfur compounds by the GC-MS system.

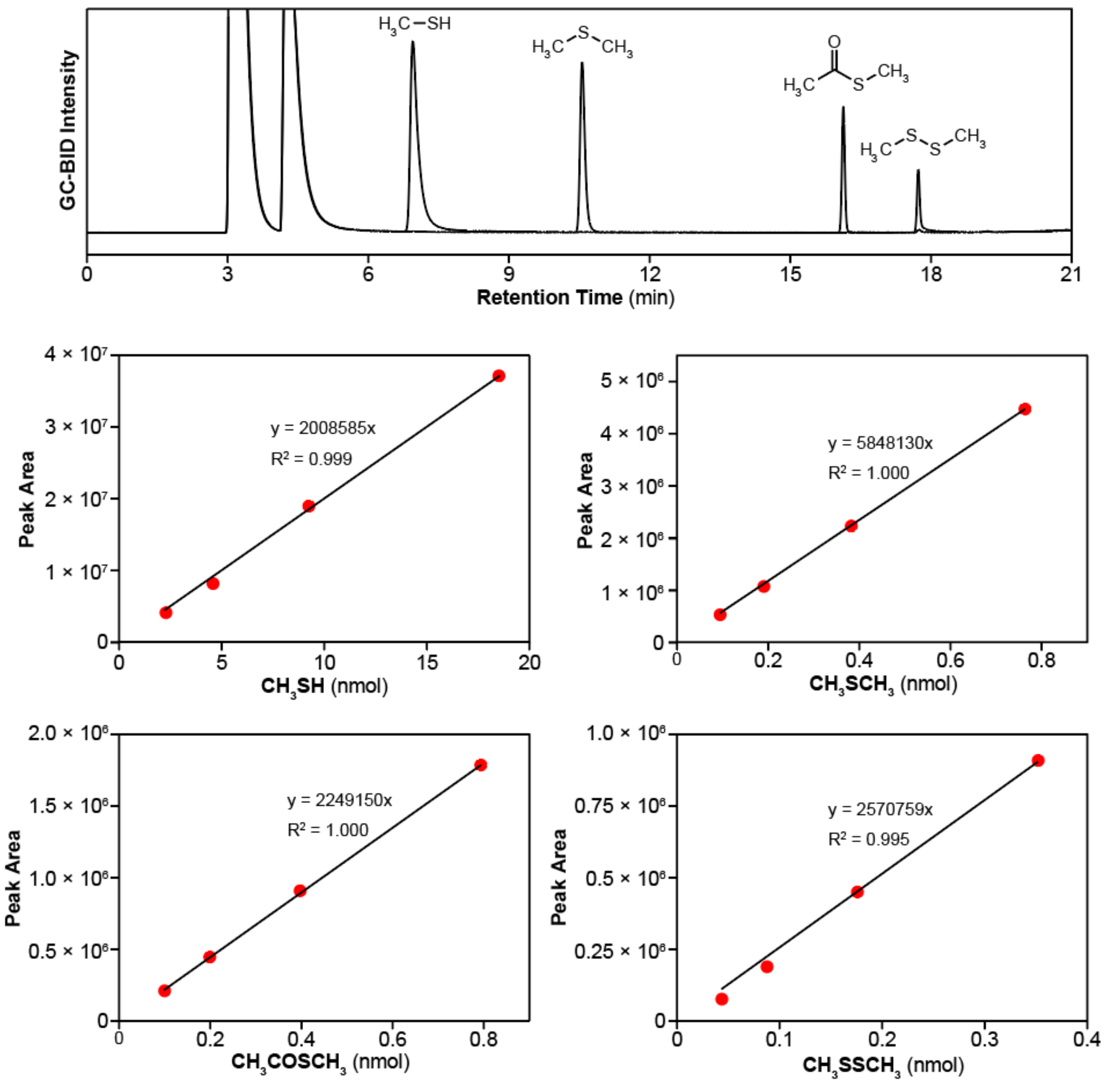

Supplementary Fig. 16 Calibration curves for organosulfur compounds by the GC-BID system.

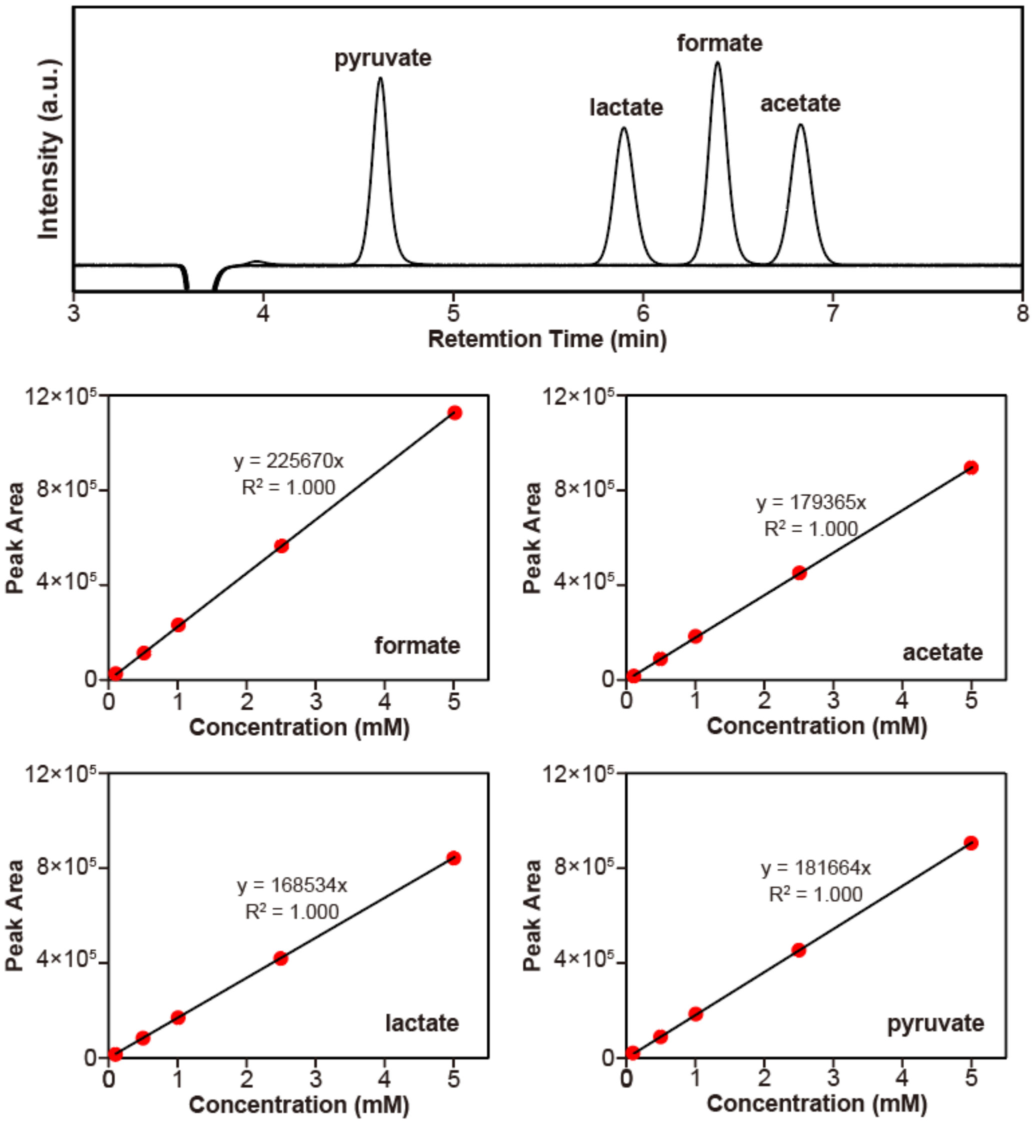

Supplementary Fig. 17 Calibration curves for organic acids by the LC-electric conductivity detector system.

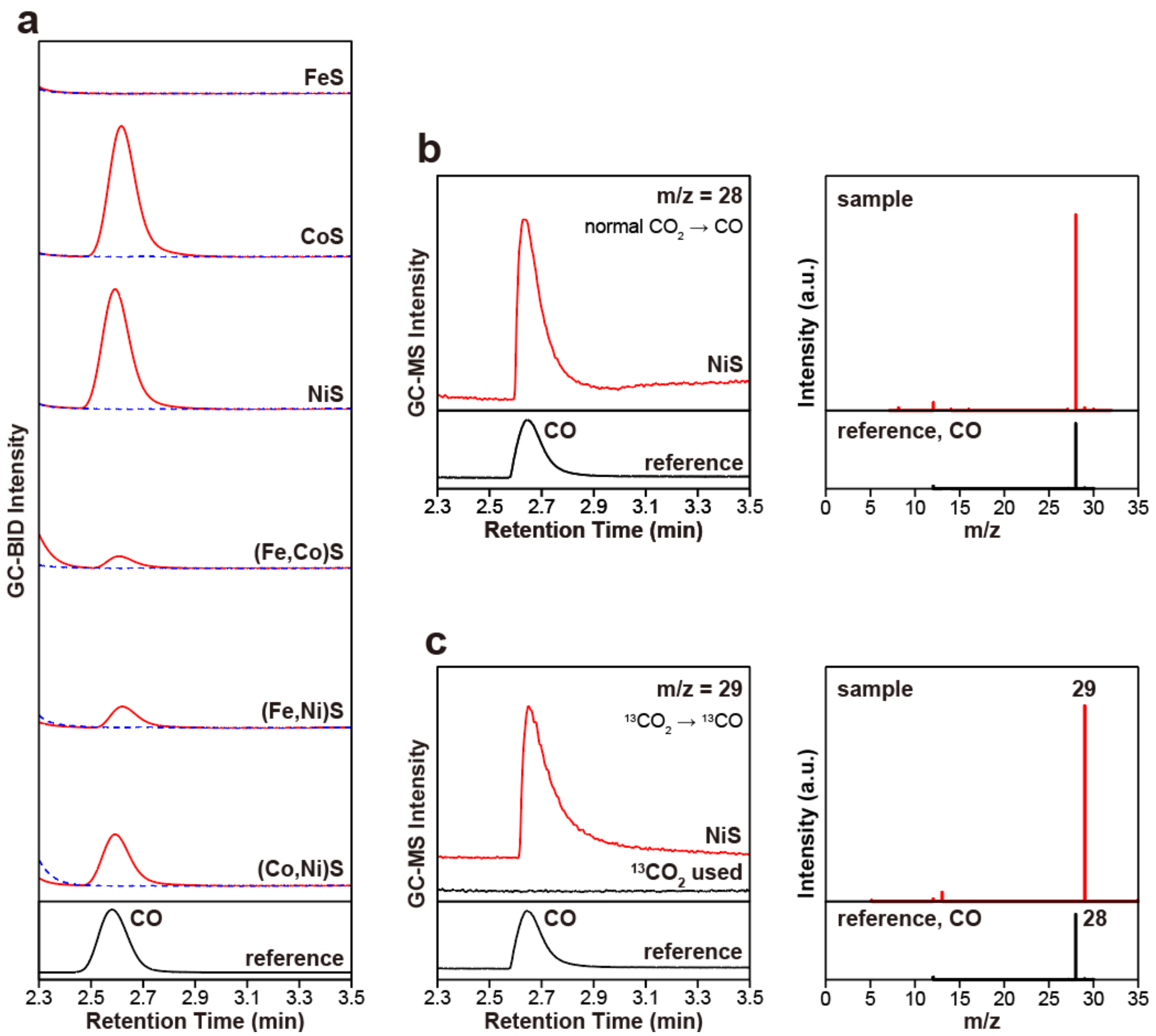

Supplementary Fig. 18 a Gas chromatograms for CO released from the metal-sulfide_PERMs prepared at –1.0 V_{SHE} (red) by complete dissolution in 35% HCl. No CO signal was observed in the same experiment using non-electrolyzed sulfides (blue). **b** and **c** GC-MS signals for the CO produced during the NiS_PERM formation under (**b**) normal and (**c**) isotopically labeled CO_2.

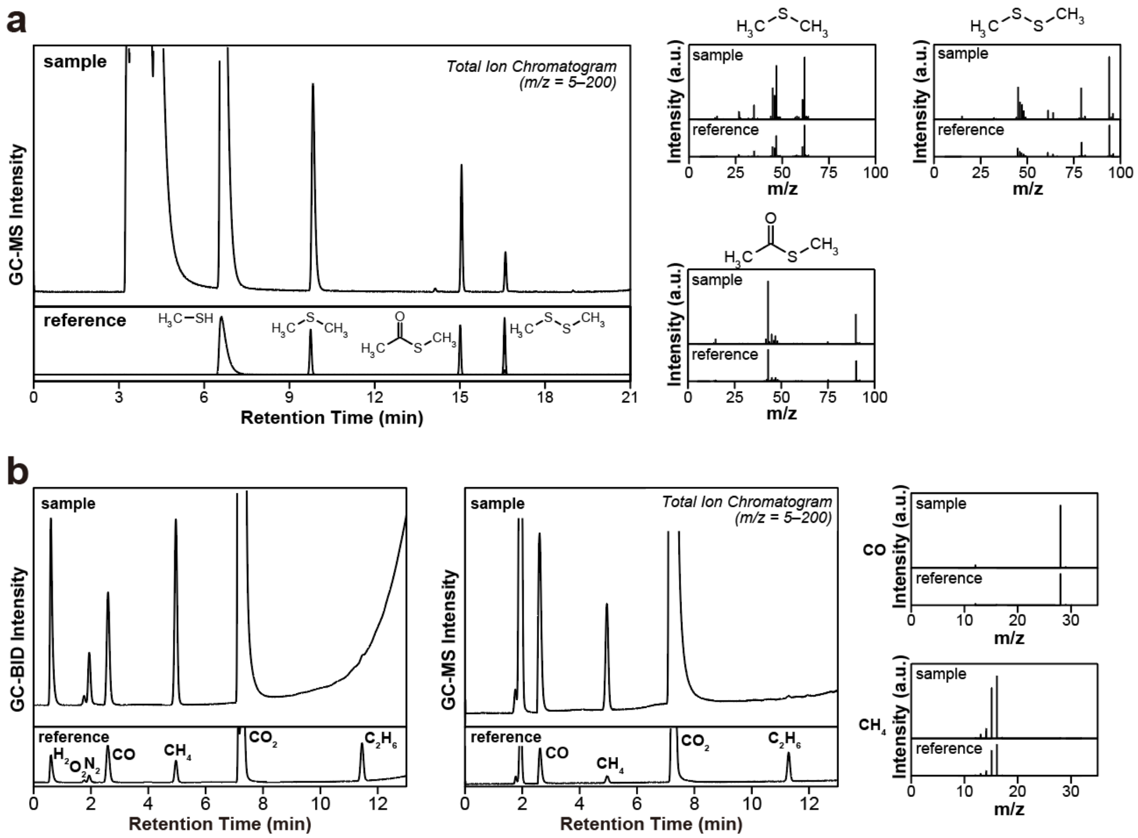

Supplementary Fig. 19 Gas chromatography characterization of the 7-day interaction of methanethiol (75 μmol) with the NiS_PERM prepared at −1.0 V_{SHE} (50 mg) at room temperature (25 ± 2°C). **a** GC-MS chromatogram for organosulfur compounds. **b** GC-BID (left) and GC-MS (right) chromatograms for inorganic gases. MTA, dimethyl sulfide, dimethyl disulfide, CO and CH_4 were identified from comparisons of retention time and mass spectrum with the corresponding standards. The broad signal at 3–5 min in the GC-MS chromatogram (**a**) is due to inorganic gas mixture including CO_2 as a dominant component with H_2O, CO, N_2 and O_2. N_2 and O_2 were always observed because of the intrusion of air into the GC systems at the sample injections.

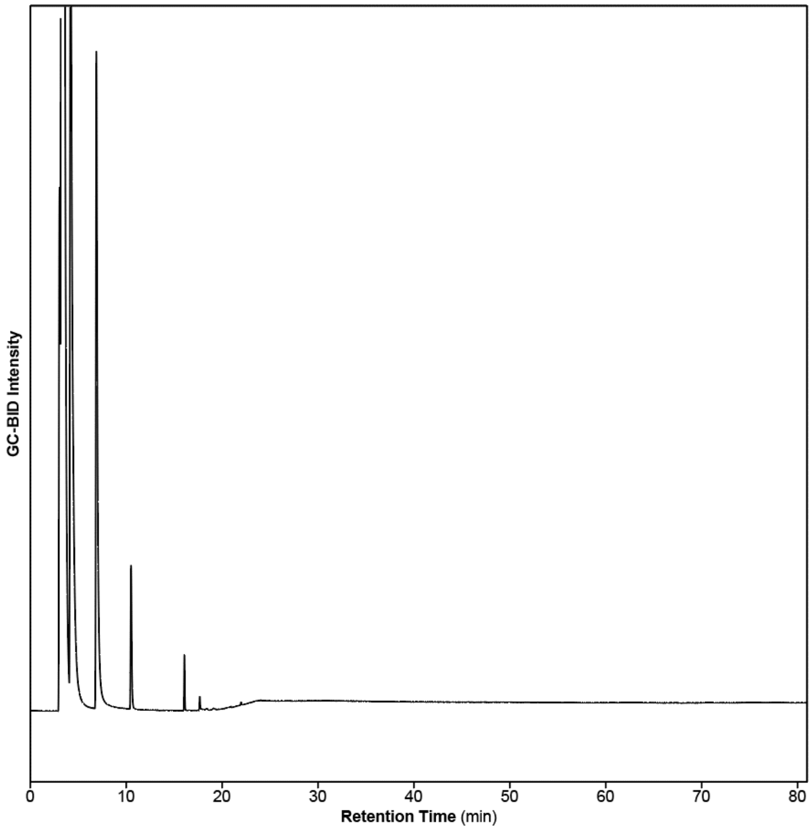

Supplementary Fig. 20 Gas sample presented in Supplementary Fig. 19 was analyzed with an extended GC time program for organosulfur compound analysis, where the highest temperature (235°C) was kept for the additional 60 min. No compounds other than methanethiol (~6.9 min), dimethyl sulfide (~10.6 min), MTA (~16.1 min), and dimethyl disulfide (~17.7 min) were observed with significant amounts (>0.01 μmol). The GC-BID chromatogram is shown here because the BID detects any species except for helium (He) and neon (Ne).

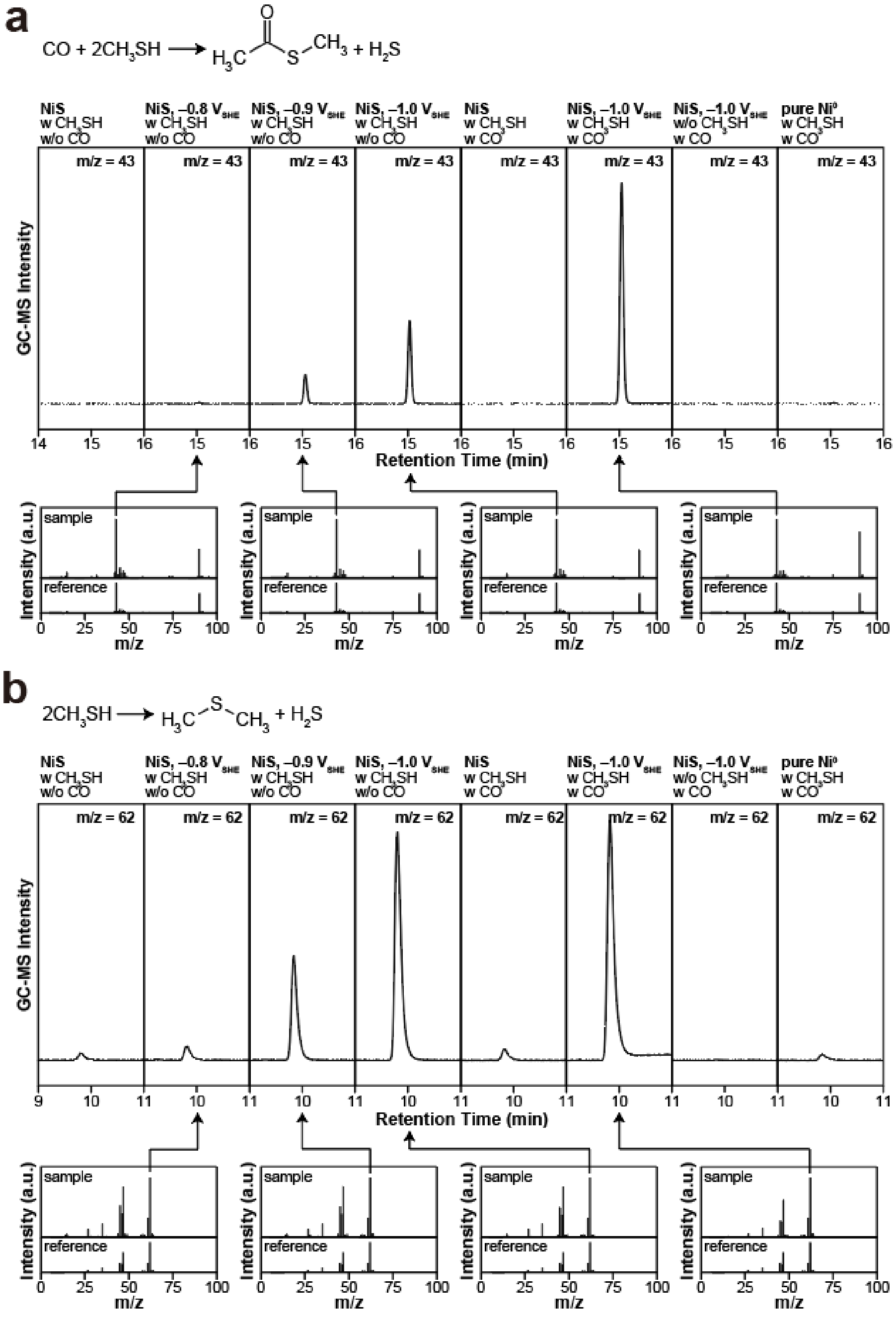

Supplementary Fig. 21 Representative GC-MS chromatograms for (**a**) MTA and (**b**) dimethyl sulfide formed at the conditions described above the respective data. All experiments were conducted at room temperature (25 ± 2°C) for 7 days. Abbreviations. w and w/o CO, with and without CO externally added (75 μmol).

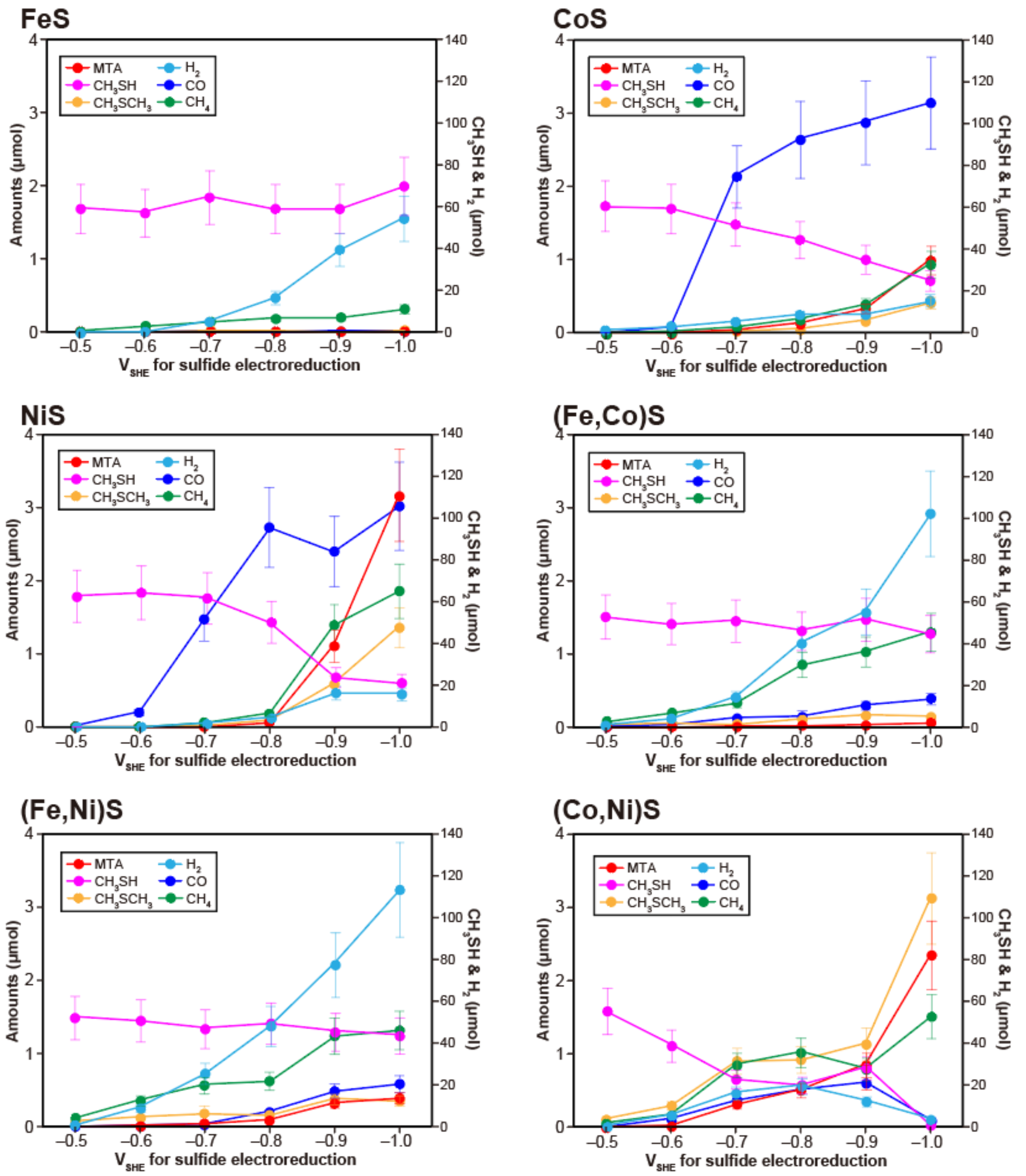

Supplementary Fig. 22 Chemicals observed after the 7-day interaction of methanethiol (initial amount; 75 μmol) with the electrolyzed sulfides (50 mg for each) at room temperature (25 ± 2°C). Dimethyl disulfide is not shown here because it is likely formed from an air oxidation of methanethiol, rather than from the examined reaction systems (see Methods). The error bars were determined based on multiple independent runs under the same reaction conditions (see Methods).

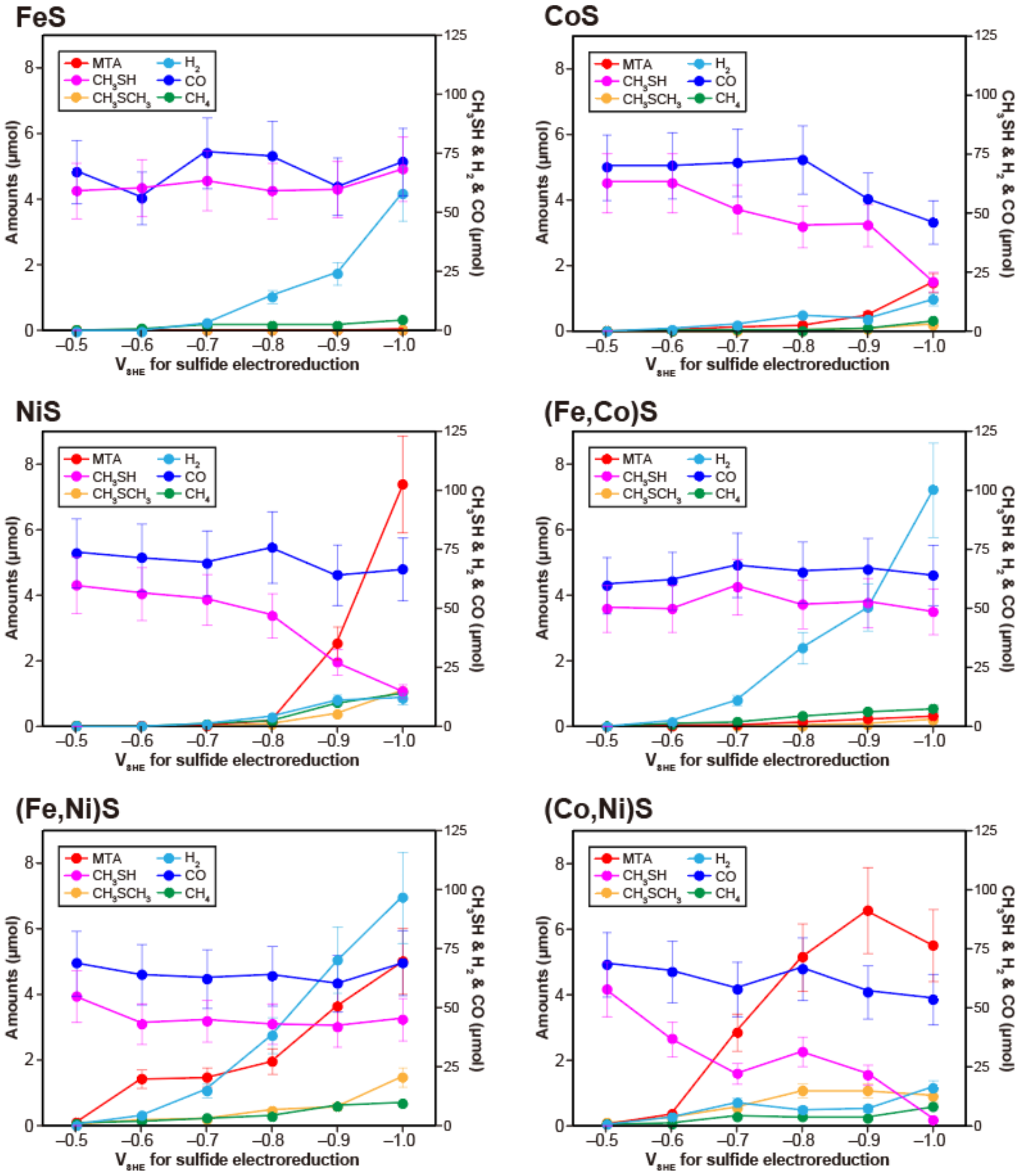

Supplementary Fig. 23 Chemicals observed after the 7-day interactions of methanethiol (initial amount; 75 μmol), CO (initial amount; 75 μmol), and the electrolyzed sulfides (50 mg for each) at room temperature (25 ± 2°C). Dimethyl disulfide is not shown here because it is likely formed from an air oxidation of methanethiol, rather than from the examined reaction systems (see Methods). The error bars were determined based on multiple independent runs under the same reaction conditions (see Methods).

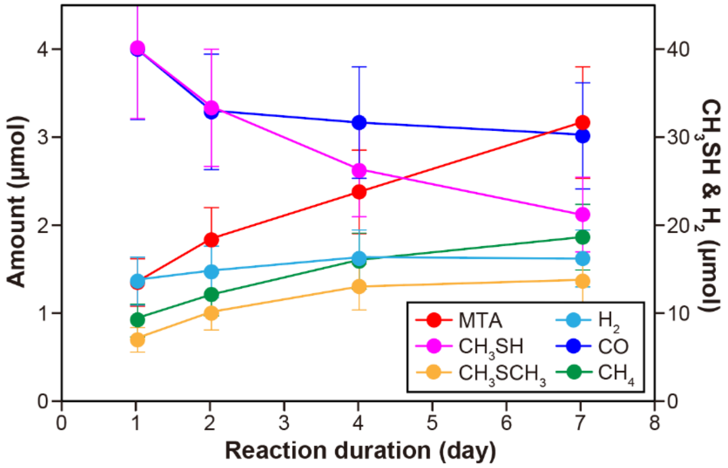

Supplementary Fig. 24 Chemicals observed after the interaction of methanethiol (initial amount; 75 μmol) and the NiS_PERM prepared at –1.0 V_{SHE} (50 mg) for 1 to 7 days at room temperature (25 ± 2°C). The error bars were determined based on multiple independent runs under the same reaction conditions (see Methods).

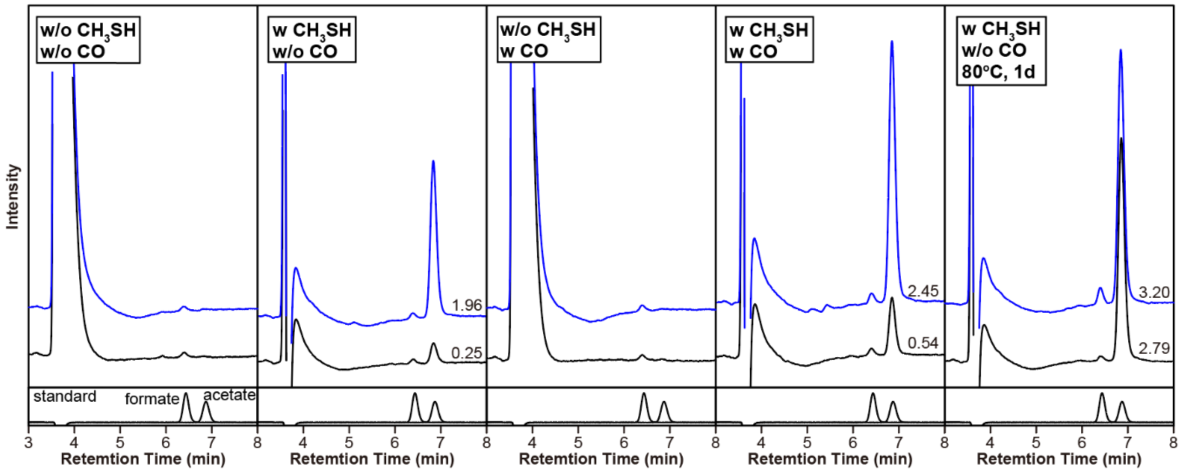

Supplementary Fig. 25 Representative HPLC data for acetate and formate formed in the presence of the NiS_PERM prepared at –1.0 V_{SHE} (50 mg) with (w) or without (w/o) CO and methanethiol externally added (75 μmol). Except for the right end chromatograms, all data were obtained after the 7-day experiments at room temperature (25 ± 2°C). Blue chromatographs were obtained after basifying the aqueous samples by 1 M NaOH.

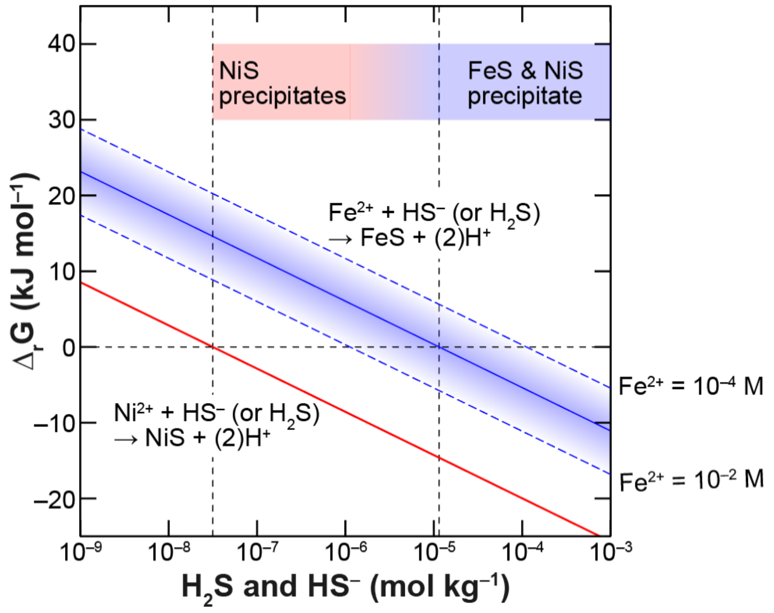

Supplementary Fig. 26 Gibbs energies of reaction for the FeS (mackinawite) and NiS (millerite) formations as a function of hydrogen sulfide (H_2S and HS^-) concentration at pH 6, 25°C, 500 bar, and the ionic strength of 0.5. Dissolved Fe^{2+} and Ni^{2+} concentrations in the early Archean seawater are estimated by Song et al.[6] (10^{-2}–10^{-4} M) and Konhauser et al.[7,8] (4×10^{-7} M), respectively.

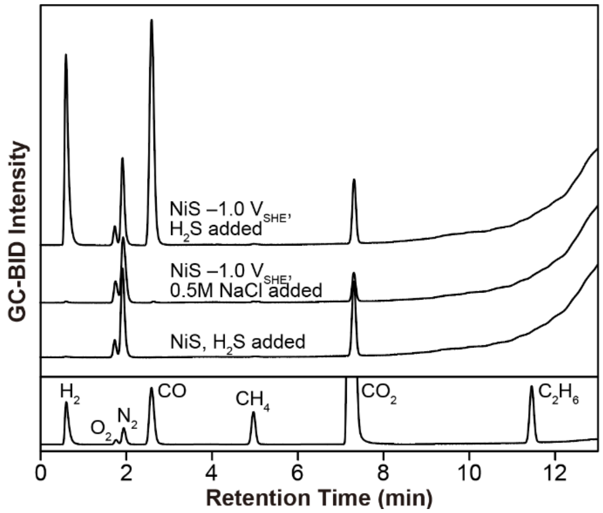

Supplementary Fig. 27 Representative GC-BID chromatograms for inorganic gases released from the NiS samples by the addition of H_2S or NaCl aqueous solution.

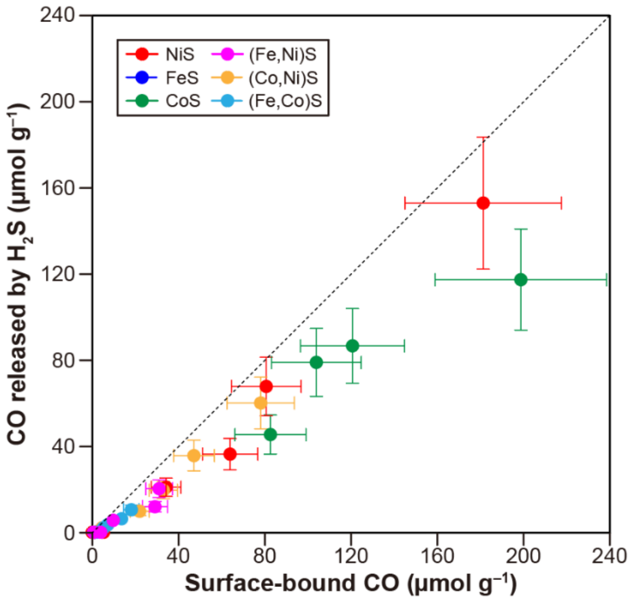

Supplementary Fig. 28 Amounts of CO released from the electrolyzed sulfides through competitive adsorption with H_2S at pH 7.5–8.0. These values are shown in comparison to the total amounts of CO on the respective sulfides (Fig. 2a and Supplementary Table 2). The error bars were determined based on multiple independent runs under the same reaction conditions (see Methods).

Supplementary Table 1 Characteristics of pure Fe^0, Co^0, and Ni^0 (EM Japan) used in the present study.

	Catalog No.	mean particle diameter	purity (%)	specific surface area (m^2 g^{-1})
Fe^0	NP-FE-2-25	95–105 nm	>99.5	4–6
Co^0	NP-CO-1-25	1.3 μm	99.9	not reported
Ni^0	NP-NI-2-5	100 nm	99.9	22.76

Supplementary Table 2 Amounts of CO released from the electrolyzed sulfides by their complete dissolution in 35% HCl.

Solid	V_{SHE}	CO (μmol/g)
FeS	−	<0.1
	−0.5	<0.1
	−0.6	<0.1
	−0.7	<0.1
	−0.8	0.4 ± 0.1
	−0.9	0.4 ± 0.1
	−1.0	0.4 ± 0.1
CoS	−	<0.1
	−0.5	<0.1
	−0.6	2.9 ± 0.6
	−0.7	81.4 ± 16
	−0.8	90.5 ± 18
	−0.9	120 ± 24
	−1.0	198 ± 40
NiS	−	<0.1
	−0.5	<0.1
	−0.6	4.7 ± 0.9
	−0.7	34.2 ± 6.8
	−0.8	64.0 ± 13
	−0.9	80.8 ± 16
	−1.0	181 ± 36
(Fe,Co)S	−	<0.1
	−0.5	<0.1
	−0.6	2.1 ± 0.4
	−0.7	5.1 ± 1.0
	−0.8	6.9 ± 1.4
	−0.9	13.4 ± 2.7
	−1.0	17.6 ± 3.5
(Fe,Ni)S	−	<0.1
	−0.5	<0.1
	−0.6	0.4 ± 0.1
	−0.7	3.5 ± 0.7
	−0.8	9.8 ± 2.0
	−0.9	29.0 ± 5.8
	−1.0	29.1 ± 5.8
(Co,Ni)S	−	<0.1
	−0.5	<0.1
	−0.6	4.5 ± 0.9
	−0.7	34.8 ± 7.0
	−0.8	45.5 ± 9.1
	−0.9	79.5 ± 16
	−1.0	92.6 ± 19

Supplementary Table 3 pH and observed chemicals after the 7-day interactions of methanethiol (initial amount; 75 μmol) and the electrolyzed sulfides (50 mg for each) at room temperature (25 ± 2°C).

Solid	V_{SHE}	pH	Chemicals (μmol)					
			H_2	CO	CH_4	**MTA**	CH_3SH	CH_3SCH_3
FeS	−	6.87	0.04 ± 0.01	<0.01	<0.01	**<0.01**	71.9 ± 14	0.02 ± 0.00
	−0.5	7.01	0.05 ± 0.01	<0.01	<0.01	**<0.01**	59.5 ± 12	<0.01
	−0.6	7.04	0.21 ± 0.04	<0.01	0.08 ± 0.02	**<0.01**	57.3 ± 11	<0.01
	−0.7	7.11	5.28 ± 1.0	<0.01	0.14 ± 0.03	**<0.01**	64.8 ± 13	0.02 ± 0.00
	−0.8	7.04	16.6 ± 0.3	<0.01	0.19 ± 0.04	**<0.01**	59.0 ± 13	<0.01
	−0.9	7.12	39.4 ± 0.8	0.02 ± 0.00	0.19 ± 0.04	**<0.01**	59.1 ± 13	<0.01
	−1.0	7.16	54.9 ± 11	0.02 ± 0.00	0.31 ± 0.06	**<0.01**	69.9 ± 14	0.02 ± 0.00
CoS	−	7.06	1.07 ± 0.21	<0.01	<0.01	**<0.01**	55.4 ± 11	<0.01
	−0.5	7.06	0.88 ± 0.18	<0.01	<0.01	**<0.01**	60.1 ± 12	<0.01
	−0.6	7.08	2.68 ± 0.54	0.08 ± 0.02	0.02 ± 0.00	**<0.01**	59.3 ± 12	<0.01
	−0.7	7.24	0.85 ± 0.17	2.15 ± 0.43	0.09 ± 0.02	**0.06 ± 0.01**	51.3 ± 10	0.04 ± 0.01
	−0.8	7.00	9.57 ± 1.9	2.87 ± 0.57	0.29 ± 0.06	**0.15 ± 0.03**	33.8 ± 6.8	0.07 ± 0.01
	−0.9	7.16	8.29 ± 1.7	2.89 ± 0.58	0.40 ± 0.08	**0.34 ± 0.07**	34.5 ± 6.9	0.18 ± 0.04
	−1.0	7.18	14.8 ± 3.0	3.14 ± 0.63	0.95 ± 0.19	**1.01 ± 0.20**	24.5 ± 4.9	0.42 ± 0.08
NiS	−	7.09	0.13 ± 0.01	<0.01	<0.01	**<0.01**	1.9 ± 0.4	0.04 ± 0.01
	−0.5	7.12	0.05 ± 0.01	<0.01	<0.01	**<0.01**	62.7 ± 13	<0.01
	−0.6	7.06	0.12 ± 0.02	0.20 ± 0.0	<0.01	**<0.01**	64.7 ± 13	<0.01
	−0.7	7.11	1.79 ± 0.36	1.48 ± 0.30	0.06 ± 0.01	**0.01 ± 0.00**	62.3 ± 13	0.02 ± 0.00
	−0.8	7.23	4.67 ± 0.93	2.73 ± 0.55	0.19 ± 0.04	**0.06 ± 0.01**	50.5 ± 10	0.09 ± 0.02
	−0.9	7.18	16.4 ± 3.3	2.40 ± 0.48	1.40 ± 0.28	**1.11 ± 0.22**	23.8 ± 4.8	0.59 ± 0.12
	−1.0	7.16	16.2 ± 3.2	3.02 ± 0.60	1.86 ± 0.37	**3.16 ± 0.62**	21.2 ± 4.2	1.37 ± 0.27
	−1.0*	6.59	10.3 ± 2.1	0.03 ± 0.01	<0.01	**<0.01**	<0.01	<0.01
(Fe,Co)S	−	7.00	0.06 ± 0.01	<0.01	0.04 ± 0.01	**<0.01**	56.8 ± 11	0.03 ± 0.01
	−0.5	7.06	1.14 ± 0.23	<0.01	0.07 ± 0.01	**<0.01**	53.0 ± 11	0.03 ± 0.01
	−0.6	7.05	4.34 ± 0.87	0.04 ± 0.01	0.19 ± 0.04	**<0.01**	49.6 ± 10	0.05 ± 0.01
	−0.7	7.20	14.6 ± 2.9	0.13 ± 0.03	0.33 ± 0.07	**0.01 ± 0.00**	51.3 ± 10	0.04 ± 0.01
	−0.8	7.07	40.4 ± 8.1	0.15 ± 0.03	0.85 ± 0.17	**0.02 ± 0.00**	46.6 ± 9.3	0.11 ± 0.02
	−0.9	7.24	55.4 ± 11	0.30 ± 0.06	1.03 ± 0.21	**0.03 ± 0.01**	51.9 ± 10	0.17 ± 0.03
	−1.0	7.18	102.4 ± 20	0.39 ± 0.08	1.31 ± 0.26	**0.06 ± 0.01**	44.8 ± 9.0	0.14 ± 0.02
(Fe,Ni)S	−	6.91	0.02 ± 0.00	<0.01	<0.01	**<0.01**	65.4 ± 13	0.03 ± 0.01
	−0.5	6.97	0.74 ± 0.15	<0.01	0.12 ± 0.02	**<0.01**	52.3 ± 10	0.08 ± 0.02
	−0.6	7.04	8.91 ± 0.18	<0.01	0.36 ± 0.07	**0.02 ± 0.00**	50.6 ± 10	0.13 ± 0.03
	−0.7	7.06	33.5 ± 6.7	0.11 ± 0.02	0.26 ± 0.05	**0.03 ± 0.01**	52.3 ± 10	0.09 ± 0.02
	−0.8	7.09	48.1 ± 9.6	0.21 ± 0.06	0.63 ± 0.13	**0.09 ± 0.02**	49.4 ± 10	0.16 ± 0.03
	−0.9	7.30	77.5 ± 16	0.49 ± 0.10	1.25 ± 0.25	**0.32 ± 0.06**	45.6 ± 9.1	0.38 ± 0.08
	−1.0	7.19	113.4 ± 23	0.58 ± 0.12	1.32 ± 0.26	**0.39 ± 0.08**	43.6 ± 8.7	0.35 ± 0.07
(Co,Ni)S	−	7.10	0.07 ± 0.01	<0.01	<0.01	**<0.01**	55.8 ± 11	<0.01
	−0.5	7.02	0.76 ± 0.15	<0.01	0.06 ± 0.01	**<0.01**	55.5 ± 11	0.11 ± 0.02
	−0.6	7.05	6.26 ± 1.3	0.12 ± 0.02	0.17 ± 0.03	**0.02 ± 0.00**	39.4 ± 7.9	0.30 ± 0.06
	−0.7	7.12	16.7 ± 3.3	0.37 ± 0.07	0.86 ± 0.17	**0.31 ± 0.06**	23.2 ± 4.6	0.90 ± 0.18
	−0.8	7.08	17.0 ± 3.4	0.84 ± 0.17	0.80 ± 0.16	**0.52 ± 0.10**	29.7 ± 5.9	0.87 ± 0.17
	−0.9	7.20	19.9 ± 4.0	1.19 ± 0.24	1.07 ± 0.21	**1.32 ± 0.26**	22.3 ± 4.5	1.26 ± 0.25
	−1.0	7.11	16.4 ± 3.3	0.50 ± 0.10	1.76 ± 0.35	**2.58 ± 0.52**	4.90 ± 0.98	2.70 ± 0.54
Pure Ni^0	−	7.02	0.36 ± 0.07	0.06 ± 0.01	0.22 ± 0.04	**<0.01**	64.5 ± 13	0.04 ± 0.01
Pure Fe^0	−	8.47	288.0 ± 58	<0.01	2.80 ± 0.56	**<0.01**	56.4 ± 11	0.05 ± 0.01
Pure Co^0	−	7.02	1.34 ± 0.27	<0.01	0.28 ± 0.06	**<0.01**	60.0 ± 12	0.06 ± 0.01

* No methanethiol was added.

Supplementary Table 4 pH and observed chemicals after the 7-day interactions of methanethiol (initial amount; 75 μmol), CO (initial amount; 75 μmol), and the electrolyzed sulfides (50 mg for each) at room temperature (25 ± 2°C).

Solid	V_{SHE}	pH	Chemicals (μmol)					
			H_2	CO	CH_4	**MTA**	CH_3SH	CH_3SCH_3
FeS	−	7.19	0.11 ± 0.02	74.6 ± 15	0.03 ± 0.01	**<0.01**	56.3 ± 11	0.03 ± 0.01
	−0.5	7.07	0.06 ± 0.01	67.6 ± 14	<0.01	**<0.01**	59.4 ± 12	<0.01
	−0.6	7.04	0.16 ± 0.03	56.5 ± 11	0.05 ± 0.01	**<0.01**	60.5 ± 12	<0.01
	−0.7	7.11	3.48 ± 0.70	75.5 ± 15	0.18 ± 0.04	**<0.01**	63.6 ± 13	0.02 ± 0.00
	−0.8	7.11	14.8 ± 3.0	74.0 ± 15	0.17 ± 0.03	**<0.01**	59.2 ± 12	<0.01
	−0.9	7.10	24.6 ± 4.9	61.0 ± 12	0.16 ± 0.03	**<0.01**	59.9 ± 12	<0.01
	−1.0	7.09	58.5 ± 11.7	71.7 ± 14	0.32 ± 0.06	**0.04 ± 0.01**	68.6 ± 14	0.02 ± 0.00
CoS	−	7.16	0.74 ± 0.15	77.1 ± 15	0.02 ± 0.00	**<0.01**	55.2 ± 11	<0.01
	−0.5	7.13	0.45 ± 0.09	70.0 ± 14	<0.01	**<0.01**	63.2 ± 13	<0.01
	−0.6	7.15	1.32 ± 0.26	70.4 ± 14	0.03 ± 0.01	**0.03 ± 0.01**	63.3 ± 13	<0.01
	−0.7	7.24	3.04 ± 0.61	71.6 ± 14	0.05 ± 0.01	**0.12 ± 0.02**	52.1 ± 10	0.02 ± 0.00
	−0.8	7.15	7.47 ± 1.5	69.2 ± 14	0.06 ± 0.01	**0.30 ± 0.06**	41.8 ± 8.4	0.04 ± 0.01
	−0.9	7.20	5.53 ± 1.2	56.1 ± 11	0.10 ± 0.02	**0.50 ± 0.10**	45.4 ± 9.1	0.08 ± 0.02
	−1.0	7.30	13.9 ± 2.8	46.3 ± 9.2	0.33 ± 0.06	**1.49 ± 0.30**	21.3 ± 0.42	0.21 ± 0.04
NiS	−	7.02	0.15 ± 0.03	68.0 ± 14	<0.01	**<0.01**	10.1 ± 2.0	0.05 ± 0.01
	−0.5	7.12	0.09 ± 0.02	73.5 ± 15	<0.01	**<0.01**	59.7 ± 12	<0.01
	−0.6	7.03	0.22 ± 0.04	71.3 ± 14	<0.01	**<0.01**	56.4 ± 11	<0.01
	−0.7	7.04	1.02 ± 0.20	69.3 ± 14	0.08 ± 0.02	**0.02 ± 0.00**	54.0 ± 11	<0.01
	−0.8	7.23	4.12 ± 0.82	75.7 ± 15	0.16 ± 0.03	**0.15 ± 0.03**	47.3 ± 9.5	0.06 ± 0.01
	−0.9	7.19	11.1 ± 2.2	64.1 ± 13	0.71 ± 0.14	**2.52 ± 0.50**	27.2 ± 5.4	0.38 ± 0.08
	−1.0	7.12	12.1 ± 2.4	66.5 ± 13	1.01 ± 0.20	**7.38 ± 1.5**	15.0 ± 3.0	1.09 ± 0.22
	−1.0*	6.39	0.91 ± 0.18	52.7 ± 11	<0.01	**<0.01**	<0.01	<0.01
(Fe,Co)S	−	7.24	0.20 ± 0.04	69.0 ± 14	0.05 ± 0.01	**<0.01**	45.8 ± 9.2	0.04 ± 0.01
	−0.5	7.06	0.35 ± 0.07	60.2 ± 12	<0.01	**<0.01**	50.5 ± 10	0.03 ± 0.01
	−0.6	7.04	2.48 ± 0.50	62.0 ± 12	0.08 ± 0.02	**0.04 ± 0.01**	50.1 ± 10	0.04 ± 0.01
	−0.7	7.20	11.4 ± 2.3	68.4 ± 14	0.15 ± 0.03	**0.06 ± 0.01**	59.5 ± 12	0.05 ± 0.01
	−0.8	7.03	33.7 ± 6.7	65.8 ± 13	0.33 ± 0.07	**0.15 ± 0.03**	51.8 ± 10	0.07 ± 0.01
	−0.9	7.24	50.8 ± 10.2	67.0 ± 13	0.46 ± 0.09	**0.24 ± 0.05**	52.8 ± 11	0.09 ± 0.02
	−1.0	7.07	100.5 ± 20.1	64.1 ± 13	0.54 ± 0.11	**0.32 ± 0.06**	49.0 ± 10	0.08 ± 0.02
(Fe,Ni)S	−	7.32	0.08 ± 0.02	61.0 ± 12	<0.01	**<0.01**	51.0 ± 10	0.04 ± 0.01
	−0.5	7.02	0.74 ± 0.15	69.1 ± 14	0.08 ± 0.02	**0.10 ± 0.02**	55.2 ± 11	0.03 ± 0.01
	−0.6	7.06	4.6 ± 0.92	64.2 ± 13	0.14 ± 0.03	**1.44 ± 0.29**	43.8 ± 8.8	0.18 ± 0.04
	−0.7	7.09	15.4 ± 3.1	62.6 ± 13	0.24 ± 0.05	**1.48 ± 0.30**	44.7 ± 8.9	0.21 ± 0.04
	−0.8	7.32	38.6 ± 7.7	64.0 ± 13	0.30 ± 0.06	**1.96 ± 0.39**	43.3 ± 8.7	0.49 ± 0.10
	−0.9	7.25	70.2 ± 14	60.7 ± 12	0.61 ± 0.12	**3.63 ± 0.73**	42.4 ± 8.5	0.59 ± 0.12
	−1.0	7.21	97.0 ± 19	69.0 ± 14	0.70 ± 0.14	**5.02 ± 1.0**	45.5 ± 9.1	1.49 ± 0.30
(Co,Ni)S	−	7.28	0.10 ± 0.02	66.7 ± 13	<0.01	**<0.01**	41.5 ± 8.3	<0.01
	−0.5	7.04	0.88 ± 0.18	68.9 ± 14	0.06 ± 0.01	**0.07 ± 0.01**	58.1 ± 12	0.10 ± 0.02
	−0.6	7.17	4.04 ± 0.81	65.8 ± 13	0.07 ± 0.01	**0.38 ± 0.08**	37.3 ± 7.5	0.26 ± 0.05
	−0.7	7.17	10.1 ± 2.0	58.3 ± 12	0.31 ± 0.06	**2.88 ± 0.58**	22.3 ± 4.5	0.56 ± 0.11
	−0.8	7.09	13.3 ± 2.7	66.6 ± 13	0.41 ± 0.08	**4.88 ± 0.98**	25.1 ± 5.0	0.93 ± 0.19
	−0.9	7.26	14.0 ± 2.8	55.7 ± 11	0.41 ± 0.08	**7.90 ± 1.6**	16.2 ± 3.2	1.19 ± 0.23
	−1.0	7.50	11.7 ± 2.3	64.3 ± 13	0.63 ± 0.13	**5.15 ± 1.0**	1.14 ± 0.23	1.10 ± 0.22
Pure Ni^0	−	6.99	0.26 ± 0.05	72.8 ± 15	0.15 ± 0.03	**0.03 ± 0.01**	62.5 ± 13	0.03 ± 0.01
Pure Fe^0	−	8.07	329.6 ± 66	75.8 ± 15	2.44 ± 0.49	**0.06 ± 0.01**	55.9 ± 11	0.05 ± 0.01
Pure Co^0	−	7.04	1.30 ± 0.26	72.8 ± 15	0.18 ± 0.04	**0.03 ± 0.01**	57.9 ± 11	0.05 ± 0.01

* No methanethiol was added.

Supplementary Table 5 pH and the gas-phase H_2 and CO quantified after the 1-day interactions of H_2S (initial amount; 300 μmol) and the electrolyzed sulfides (50 mg for each) at room temperature (25 ± 2°C).

Solid	V_{SHE}	pH	H_2 (μmol)	CO (μmol)
FeS	−	7.56	0.14 ± 0.03	<0.01
	−0.5	7.55	0.17 ± 0.03	<0.01
	−0.6	7.56	0.16 ± 0.03	<0.01
	−0.7	7.58	0.39 ± 0.08	<0.01
	−0.8	7.46	4.05 ± 0.81	<0.01
	−0.9	7.70	27.7 ± 5.5	<0.01
	−1.0	7.76	38.6 ± 7.7	<0.01
CoS	−	7.60	0.99 ± 0.20	<0.01
	−0.5	7.50	0.63 ± 0.13	<0.01
	−0.6	7.51	1.35 ± 0.27	0.03 ± 0.01
	−0.7	7.63	10.1 ± 2.0	2.79 ± 0.56
	−0.8	7.50	10.2 ± 2.0	3.94 ± 0.79
	−0.9	7.67	17.6 ± 3.5	4.34 ± 0.87
	−1.0	7.68	26.5 ± 5.3	5.88 ± 1.2
NiS	−	7.68	0.09 ± 0.02	<0.01
	−0.5	7.59	0.84 ± 0.17	<0.01
	−0.6	7.56	0.77 ± 0.15	0.03 ± 0.01
	−0.7	7.52	0.90 ± 0.18	1.06 ± 0.21
	−0.8	7.60	3.19 ± 0.64	1.83 ± 0.37
	−0.9	7.68	18.5 ± 3.7	3.41 ± 0.68
	−1.0	7.68	20.9 ± 4.2	7.64 ± 1.5
(Fe,Co)S	−	7.59	0.61 ± 0.12	<0.01
	−0.5	7.57	2.44 ± 0.49	<0.01
	−0.6	7.57	5.54 ± 1.1	0.03 ± 0.01
	−0.7	7.59	13.6 ± 2.7	0.12 ± 0.02
	−0.8	7.62	32.8 ± 6.6	0.18 ± 0.04
	−0.9	7.77	48.5 ± 9.7	0.33 ± 0.07
	−1.0	8.01	85.3 ± 17	0.52 ± 0.10
(Fe,Ni)S	−	7.56	0.10 ± 0.02	<0.01
	−0.5	7.59	5.74 ± 1.5	<0.01
	−0.6	7.64	23.6 ± 4.7	<0.01
	−0.7	8.05	31.3 ± 6.3	0.10 ± 0.02
	−0.8	7.74	54.1 ± 11	0.31 ± 0.06
	−0.9	8.04	78.9 ± 16	0.60 ± 0.12
	−1.0	8.16	92.6 ± 19	1.04 ± 0.21
(Co,Ni)S	−	7.58	0.49 ± 0.10	<0.01
	−0.5	7.55	1.72 ± 0.34	<0.01
	−0.6	7.65	18.7 ± 3.7	0.11 ± 0.02
	−0.7	7.81	45.0 ± 9.0	0.90 ± 0.18
	−0.8	7.73	36.7 ± 7.3	1.74 ± 0.35
	−0.9	7.87	57.8 ± 12	3.64 ± 0.73
	−1.0	7.98	70.2 ± 14	4.18 ± 0.84

References for supplementary Information

1. Kitadai, N. et al. Metals likely promoted protometabolism in early ocean alkaline hydrothermal systems. *Sci. Adv.* **5**, eeav7848 (2019).
2. Krissansen-Totton, J., Arney, G. N. & Catling, D. C. Constraining the climate and ocean pH of the early Earth with a geological carbon cycle model. *Proc. Natl. Acad. Sci. USA* **115**, 4105–4110 (2018).
3. Tivey, M. K. Generation of seafloor hydrothermal vent fluids and associated mineral deposits. *Oceanography* **20**, 50–65 (2007).
4. Schrenk, M. O., Brazelton, W. J. & Lang, S. Q. Serpentinization, carbon, and deep life. *Rev. Mineral. Geochem.* **75**, 575–606 (2013).
5. Fleet, M. E. The crystal structure of heazlewoodite, and metallic bonds in sulfide minerals. *Am. Mineral.* **62**, 341–345 (1977).
6. Song, H. et al. The onset of widespread marine red beds and the evolution of ferruginous oceans. *Nat. Commun.* **8**, 399 (2017).
7. Konhauser, K. O. et al. Oceanic nickel depletion and a methanogen famine before the great oxidation event. *Nature* **458**, 750–753 (2009).
8. Konhauser, K. O. et al. The Archean nickel famine revisited. *Astrobiology* **15**, 804–815 (2015).
