# Supplementary material for: Thioester synthesis through geoelectrochemical CO2 fixation on Ni sulfides
Source: Commun Chem. 2021 Mar 17;4:37. doi: 10.1038/s42004-021-00475-5 (PMC9814748; doi:10.1038/s42004-021-00475-5)
Supplement: Supplementary file 2 — Description of Additional Supplementary Files [file 42004_2021_475_MOESM2_ESM.pdf]

Description of Additional Supplementary Files

File Name: Supplementary Movie 1
Description: Demonstration of the surface-bound CO on the NiS_PERM prepared at –1.0 V_{SHE}. 1 M H_3PO_4 was added into serum bottles containing 50 mg of pure NiS (left) or the NiS_PERM (right).
